# Flexible intentions: An Active Inference theory

**DOI:** 10.3389/fncom.2023.1128694

**Published:** 2023-03-20

**Authors:** Matteo Priorelli, Ivilin Peev Stoianov

**Affiliations:** Institute of Cognitive Sciences and Technologies (ISTC), National Research Council of Italy (CNR), Padua, Italy

**Keywords:** Active Inference, sensorimotor control, Posterior Parietal Cortex, intentions, Predictive Coding

## Abstract

We present a normative computational theory of how the brain may support visually-guided goal-directed actions in dynamically changing environments. It extends the Active Inference theory of cortical processing according to which the brain maintains beliefs over the environmental state, and motor control signals try to fulfill the corresponding sensory predictions. We propose that the neural circuitry in the Posterior Parietal Cortex (PPC) compute flexible intentions—or motor plans from a belief over targets—to dynamically generate goal-directed actions, and we develop a computational formalization of this process. A proof-of-concept agent embodying visual and proprioceptive sensors and an actuated upper limb was tested on target-reaching tasks. The agent behaved correctly under various conditions, including static and dynamic targets, different sensory feedbacks, sensory precisions, intention gains, and movement policies; limit conditions were individuated, too. Active Inference driven by dynamic and flexible intentions can thus support goal-directed behavior in constantly changing environments, and the PPC might putatively host its core intention mechanism. More broadly, the study provides a normative computational basis for research on goal-directed behavior in end-to-end settings and further advances mechanistic theories of active biological systems.

## 1. Introduction

Traditionally, sensorimotor control in goal-directed actions like object-reaching is viewed as a sensory-response mapping involving several steps, starting with perception, movement planning in the body posture domain, translation of this plan in muscle commands, and finally movement execution (Erlhagen and Schöner, 2002). However, each of these steps is hindered by noise and delays, which make the approach unfeasible to operate in changing environments (Franklin and Wolpert, 2011). Instead, Predictive Coding or “Bayesian Brain” theories propose that prior knowledge and expectations over the environmental and bodily contexts provide crucial anticipatory information (Rao and Ballard, 1999). Under this perspective, motor control begins with target anticipation and motor planning even before obtaining sensory evidence. Here, we take on this view and extend an increasingly popular Predictive Coding based theory of action, Active Inference (Friston et al., 2010), with the formalization of flexible target-dependent motor plans. Moreover, based on extensive neural evidence for the role of the PPC in goal coding and motor planning (Snyder et al., 2000; Galletti et al., 2022), we propose that this cortical structure is the most likely neural correlate of the core intention manipulation process.

In primates, the dorsomedial visual stream provides critical support for continuously monitoring the body posture and the spatial location of objects to specify and guide actions, and for performing visuomotor transformations in the course of the evolving movement (Cisek and Kalaska, 2010; Fattori et al., 2017; Galletti and Fattori, 2018). The PPC, located at the apex of the dorsal stream, is also bidirectionally connected to frontal areas, motor and somatosensory cortex, placing it in a privileged position to set goal-directed actions and continuously adjust motor plans by tracking moving targets and posture (Andersen, 1995; Gamberini et al., 2021) in a common reference frame (Cohen and Andersen, 2002). Undoubtedly, the PPC plays a crucial role in visually-guided motor control (Desmurget et al., 1999; Filippini et al., 2018; Gamberini et al., 2021)—with the specific subregion V6A involved in the control of reach-to-grasp actions (Galletti et al., 2022)—but its peculiar role is still disputed. The most consistent view is that the PPC estimates the states of both body and environment and optimizes their interactions (Medendorp and Heed, 2019). Others see the PPC as a task estimator (Haar and Donchin, 2020) or as being involved in endogenous attention and task setting (Corbetta and Shulman, 2002). Its underlying computational mechanism is not fully understood, especially as regards the definition of goals in motor planning and their integration within the control process (Shadmehr and Krakauer, 2008). For example, the prevailing Optimal Feedback Control theory defines motor goals through task-specific cost functions (Todorov, 2004). Neural-level details of motor goal coding are becoming increasingly important in light of the growing demand for neural interfaces that provide information about motor intents (Gallego et al., 2022) in support of intelligent assistive devices (Velliste et al., 2008; Srinivasan et al., 2021).

Intentions encode motor goals—or plans—set before the beginning of motor acts themselves and could be therefore viewed as memory holders of voluntary actions (Andersen, 1995; Snyder et al., 1997; Lau et al., 2004; Fogassi et al., 2005). Several cortical areas handle different aspects of this process: the Premotor cortex (PM) encodes structuring while the Supplementary Motor Area (SMA) controls phasing (Gallego et al., 2022). In turn, the PPC plays a role in building motor plans and their dynamic tuning, as different PPC neurons are sensitive to different intentions (Snyder et al., 2000). Notably, intention neurons respond not only when performing a given action but also during its observation, allowing observers to *predict the goal of the observed action and, thus, to “read” the intention of the acting individual* (Fogassi et al., 2005). Motor goals have also been observed down the motor hierarchy, which is an expression of Hierarchical Predictive Coding in the motor domain (Friston et al., 2011).

To investigate how neural circuitry in the PPC supports sensory-guided actions through motor intentions from a computational point of view, we adopted the Active Inference theory of cognitive and motor control, which provides fundamental insights of increasing appeal about the computational role and principles of the nervous system, especially about the perception-action loop (Friston and Kiebel, 2009; Friston et al., 2010; Bogacz, 2017; Parr et al., 2022). Indeed, Active Inference provides a formalization of these two cortical tasks, both of which are viewed as aiming to resolve the critical goal of all organisms: to survive in uncertain environments by operating within preferred states (e.g., maintaining a constant temperature). Accordingly, both tasks are implemented by dynamic minimization of a quantity called *free energy*, whose process generally corresponds to the minimization of high- and low-level prediction errors, that is, the satisfaction of prior and sensory expectations. There are two branches of Active Inference appropriate to tackle two different levels of control. The discrete framework can explain high-level cognitive control processes such as planning and decision-making, i.e., it evaluates expected outcomes to select actions in discrete entities (Pezzulo et al., 2018). In turn, dynamic adjustment of action plans in the PPC matches by functionality the Active Inference framework in continuous state space (Friston et al., 2011, 2012). In short, this theory departs from classical views of perception, motor planning (Erlhagen and Schöner, 2002), and motor control (Todorov, 2004), unifying and considering them as a dynamic probabilistic inference problem (Toussaint and Storkey, 2006; Kaplan and Friston, 2018; Levine, 2018; Millidge et al., 2020). The biologically implausible cost functions typical of Optimal Control theories are replaced by high-level priors defined in the extrinsic state space, allowing complex movements such as walking or handwriting (Friston, 2011; Adams et al., 2013).

In the following, we first outline the background computational framework and then elaborate on movement planning and intentionality in continuous Active Inference. Our most critical contributions regard the formalization of goal-directed behavior and the processes linking dynamic goals (e.g., moving visual targets) with motor plans through the definition of flexible intentions. We also investigate a more parsimonious approach to motor control based solely on proprioceptive predictions. We then provide implementation details and a practical demonstration of the theoretical contribution in terms of a simulated Active Inference agent, which we show is capable of detecting and reaching static visual goals and tracking moving targets. We also provide detailed performance statistics and investigate the effects of system parameters whose balance is critical to movement stability. Additionally, gradient analysis provides crucial insights into the causes of the movements performed. Finally, we discuss how intentions could be selected to perform a series of goal-directed steps, e.g., a multi-phase action, and illustrate conditions for neurological disorders.

## 2. Computational background

We first outline the computational principles of the underlying probabilistic and Predictive Coding approach and provide background on variational inference, free energy minimization, Active Inference, and variational autoencoders necessary to comprehend the following main contribution.

### 2.1. The Bayesian brain hypothesis

An interesting visual phenomenon, called *binocular rivalry*, happens when two different images are presented simultaneously to each eye: the perception does not conform to the visual input but alternates between the two images. How and why does this happen? It is well-known that priors play a fundamental role in driving the dynamics of perceptual experience, but dominant views of the brain as a feature detector that passively receives sensory signals and computes motor commands have so far failed to explain how such illusions could arise.

In recent years, there has been increasing attention to a radically new theory of the mind called the *Bayesian brain*, according to which our brain is a sophisticated machine that constantly makes use of Bayesian reasoning to capture causal relationships in the world and deliver optimal behavior in an uncertain environment (Doya, 2007; Hohwy, 2013; Pezzulo et al., 2017). At the core of the theory is the Bayes theorem, whose application here implies that posterior beliefs about the world are updated according to the product of prior beliefs and the likelihood of observing sensory input. In this view, perception is more than a simple bottom-up feedforward mechanism that detects features and objects from the current sensorium; rather, it comprises a predictive top-down generative model which continuously anticipates the sensory input to test hypotheses and explain away ambiguities.

According to the Bayesian brain hypothesis, this complex task is accomplished by *Predictive Coding*, implemented through message passing of top-down predictions and bottom-up prediction errors between adjacent cortical layers (Rao and Ballard, 1999). The former are generated from latent states maintained at the highest levels, representing beliefs about the causes of the environment, while the latter are computed by comparing sensory-level predictions with the actual observations. Each prediction will then act as a cause for the layer below, while the prediction error will convey information to the layer above. It is thanks to this hierarchical organization and through error minimization at every layer that the cortex is supposed to be able to mimic and capture the inherently hierarchical relationships that model the world. In this view, sensations are only needed in that they provide, through the computation of prediction errors, a measure of how good the model is and a cue to correct future predictions. Thus, ascending projections do not encode the features of a stimulus, but rather how much the brain is *surprised* about it, considering the strict correlation between surprise and model uncertainty.

### 2.2. Variational bayes

Organisms are supposed to implement model fit or error minimization by some form of variational inference, a broad family of techniques based on the *calculus of variations* and used to approximate intractable posteriors that would otherwise be infeasible to compute analytically or even with classical sampling methods like Monte Carlo (Bishop, 2006). Under the Bayesian brain hypothesis, we can assume that the nervous system maintains latent variables ***z*** about both the unknown state of the external world and the internal state of the organism. By exploiting a prior knowledge *p*(***z***) and the partial evidence *p*(***s***) of the environment provided by its sensors, it can apply Bayesian inference to improve its knowledge (Ma et al., 2006). To do so, given the observation ***s***, the nervous system needs to evaluate the posterior *p*(***z***|***s***):


(1) $$\begin{array}{l} p\left(z|s\right)=\frac{p\left(z,s\right)}{p\left(s\right)} \end{array}$$

However, directly computing such quantity is infeasible due to the intractability of the marginal *p*(***s***) = ∫*p*(***z, s***)*d**z***, which involves integration over the joint density *p*(***z, s***). What does the variational approach is approximating the posterior with a simpler to compute *recognition* distribution *q*(***z***)≈*p*(***z***|***s***) through minimization of the Kullback-Leibler (KL) divergence between them:


(2) $$\begin{array}{l} D_{KL}\left[q\left(z\right)||p\left(z|s\right)\right]=\int_{z}q\left(z\right)\ln\;\frac{q\left(z\right)}{p\left(z|s\right)}dz \end{array}$$

The KL divergence can be rewritten as the difference between log evidence ln *p*(***s***) and a quantity L(q) known as *evidence lower bound*, or ELBO (Bishop, 2006):


(3) $$\begin{array}{l} D_{KL}\left[q\left(z\right)||p\left(z|s\right)\right]=\ln\;p\left(s\right)-\int_{z}q\left(z\right)\ln\;\frac{p\left(z,s\right)}{q\left(z\right)}dz=\ln\;p\left(s\right)-L\left(q\right) \end{array}$$

Since the KL divergence is always nonnegative, the ELBO provides a lower bound on log evidence, i.e., L(q)≤ln p(s). Therefore, minimizing the KL divergence with respect to *q*(***z***) is equivalent to maximizing L(q), which at its maximum corresponds to an approximate density that is closest the most to the real posterior, depending on the particular choice of the form of *q*(***z***). In general, few assumptions are made about the form of this distribution—a multivariate Gaussian is a typical choice—with a trade-off between having a tractable optimization process and still leading to a good approximate posterior.

### 2.3. Free energy and prediction errors

How can Bayesian inference be implemented through a simple message passing of prediction errors? Friston (2002, 2005) proposed an elegant solution based on the so-called *free energy*, a concept borrowed from thermodynamics and defined as the negative ELBO. Accordingly, Equation (3) can be rewritten as:


(4) $$\begin{array}{l} F\left(z,s\right)=-L\left(q\right)=D_{KL}\left[q\left(z\right)||p\left(z|s\right)\right]-\ln\;p\left(s\right)=\underset{z}{\int }q\left(z\right)\ln\;\frac{q\left(z\right)}{p\left(z,s\right)}dz \end{array}$$

Minimizing the free energy with respect to the latent states ***z***—a process called *perceptual inference*—is then equivalent to ELBO maximization and provides an upper bound on surprise:


(5) $$\begin{array}{l} z=\underset{z}{\text{arg min }}F\left(z,s\right) \end{array}$$

In this way, the organism indirectly minimizes model uncertainty and is able to learn the causal relations between unknown states and sensory input, and to generate predictions based on its current representation of the environment. Free energy minimization is simpler than dealing with the KL divergence between the approximate and true posteriors as the former depends on quantities that the organism has access to, namely the approximate posterior and the *generative model*.

To this concern, it is necessary to distinguish between the latter and the real distribution producing sensory data, called *generative process*, which can be modeled with the following non-linear stochastic equations:


(6) $$\begin{array}{l} \begin{array}{c} s=g\left(z\right)+w_{s}\\ \overset{\cdot }{z}=f\left(z\right)+w_{z} \end{array} \end{array}$$

Where the function ***g*** maps latent states or causes ***z*** to observed states or sensations ***s***, the function ***f*** encodes the dynamics of the system, i.e., the evolution of ***z*** over time, while ***w***_*s*_ and ***w***_*z*_ are noise terms that describe system uncertainty.

Nervous systems are supposed to approximate the generative process by making a few assumptions: that (i) under the mean-field approximation the recognition density can be partitioned into independent distributions: *q*(***z***) = ∏_*i*_*q*(***z***_*i*_), and that (ii) under the Laplace approximation each of these partitions is Gaussian: $q\left(z_{i}\right)=N\left(\mu_{i},\Pi_{i}^{-1}\right)$, where ***μ***_*i*_ represents the most plausible hypothesis—also called *belief* about the hidden state ***z***_*i*_ - and **Π**_*i*_ is its precision matrix (Friston et al., 2007). In this way, the free energy does not depend on *z* and simplifies as follows:


(7) $$\begin{array}{l} F\left(\mu ,s\right)=-\ln\;p\left(\mu ,s\right)+C=-\ln\;p\left(s|\mu \right)-\ln\;p\left(\mu \right)+C \end{array}$$

Where *C* is a constant term. A more precise description of the unknown environmental dynamics can be achieved by considering not only the 1st order of Equation 6 but also higher temporal orders of the corresponding approximations: $\tilde{\mu }=\left\{\mu ,\mu^{\prime },\mu^{\prime \prime },...\right\}$—called *generalized coordinates* (Friston, 2008; Friston et al., 2008). This allows us to better represent the environment with the following generalized model:


(8) $$\begin{array}{l} \begin{array}{c} \overset{\sim }{s}=\overset{\sim }{g}\left(\overset{\sim }{\mu }\right)+w_{s}\\ D\overset{\sim }{\mu }=\overset{\sim }{f}\left(\overset{\sim }{\mu }\right)+w_{\mu } \end{array} \end{array}$$

Where D is the differential (shift) operator matrix such that $D\tilde{\mu }=\left\{\mu^{\prime },\mu^{\prime \prime },...\right\}$, $\tilde{s}$ denotes the generalized sensors, while $\tilde{g}$ and $\tilde{f}$ denote the generalized model functions of all temporal orders. Note that in this system, the sensory data at a particular dynamical order ***s***^[*d*]^—where [*d*] is the order—engage only with the same order of belief ***μ***^[*d*]^, while the generalized equation of motion, or system dynamics, specifies the coupling between adjacent orders. Such equations are generated from the generalized likelihood and prior distributions, which can be expanded as follows:


(9) $$\begin{array}{l} \begin{array}{l} p\left(\tilde{s}|\tilde{\mu }\right)=\underset{d}{\prod }p\left(s^{\left[d\right]}|\mu^{\left[d\right]}\right)\\ \;p\left(\tilde{\mu }\right)=\underset{d}{\prod }p\left(\mu^{\left[d+1\right]}|\mu^{\left[d\right]}\right) \end{array} \end{array}$$

As defined above, these variational probability distributions are assumed to be Gaussian:


(10) $$\begin{array}{l} \begin{array}{l} \;p\left(s^{\left[d\right]}|\mu^{\left[d\right]}\right)=\frac{\Pi_{s}}{\sqrt{{\left(2\pi \right)}^{L}}}\exp\left(-\frac{1}{2}{\varepsilon_{s}^{\left[d\right]}}^{T}\Pi_{s}\varepsilon_{s}^{\left[d\right]}\right)\\ p\left(\mu^{\left[d+1\right]}|\mu^{\left[d\right]}\right)=\frac{\Pi_{\mu }}{\sqrt{{\left(2\pi \right)}^{M}}}\exp\left(-\frac{1}{2}{\varepsilon_{\mu }^{\left[d\right]}}^{T}\Pi_{\mu }\varepsilon_{\mu }^{\left[d\right]}\right) \end{array} \end{array}$$

Where *L* and *M* are the dimensions of sensations and internal beliefs, respectively with precisions **Π**_*s*_ and **Π**_μ_. Note that the probability distributions are expressed in terms of sensory and dynamics prediction errors:


(11) $$\begin{array}{l} \varepsilon_{s}^{\left[d\right]}=s^{\left[d\right]}-g^{\left[d\right]}\left(\mu^{\left[d\right]}\right) \end{array}$$

(12) $$\begin{array}{l} \varepsilon_{\mu }^{\left[d\right]}=\mu^{\left[d+1\right]}-f^{\left[d\right]}\left(\mu^{\left[d\right]}\right) \end{array}$$

The factorized probabilistic approximation of the dynamic model allows easy state estimation performed by iterative gradient descent over the generalized coordinates, that is, by changing the belief $\tilde{\mu }$ over the hidden states at every temporal order:


(13) $$\begin{array}{l} \overset{\cdot }{\overset{\sim }{\mu }}-D\overset{\sim }{\mu }=-\partial_{\overset{\sim }{\mu }}F\left(\overset{\sim }{\mu },\overset{\sim }{s}\right) \end{array}$$

Gradient descent is tractable because the Gaussian variational functions are smooth and differentiable and the derivatives are easily computed in terms of generalized prediction errors, since the logarithm of Equation (7) vanishes the exponent of the Gaussian. The belief update thus turns to:


(14) $$\begin{array}{l} \overset{\cdot }{\tilde{\mu }}=D\tilde{\mu }+{\frac{\partial \tilde{g}}{\partial \tilde{\mu }}}^{T}{\tilde{\Pi }}_{s}{\tilde{\varepsilon }}_{s}+{\frac{\partial \tilde{f}}{\partial \tilde{\mu }}}^{T}{\tilde{\Pi }}_{\mu }{\tilde{\varepsilon }}_{\mu }-D^{T}{\tilde{\Pi }}_{\mu }{\tilde{\varepsilon }}_{\mu } \end{array}$$

It is crucial to keep in mind the nature of the three components that compose this update equation: a likelihood error computed at the sensory level, a backward error arising from the next temporal order, and a forward error coming from the previous order. These terms represent the free energy gradients relative to the belief ***μ***^[*d*]^ of Equation (11) for the likelihood, and ***μ***^[*d*+1]^ and ***μ***^[*d*]^ of Equation (12) for the dynamics errors.

In short, by making a few plausible simplifying assumptions, the complexity of free energy minimization reduces to the generation of predictions, which are constantly compared with sensory observations to determine a prediction error signal. This error then flows back through the cortical hierarchy to adjust the distribution parameters accordingly and minimize sensory surprise—or maximize evidence—in the long run.

### 2.4. Active Inference

Describing the relationship between Predictive Coding and Bayesian inference still does not explain why has the cortex evolved in such a peculiar way. The answer comes from the so-called *free energy principle* (FEP), regard to which the Bayesian brain hypothesis is supposed to be a corollary. Indeed, learning the causal relationships of some observed data (e.g., what causes an increase in body temperature) is insufficient to keep organisms alive (e.g., maintaining the temperature in a vital range).

The FEP states that, for an organism to maintain a state of homeostasis and survive, it must constantly and actively restrict the set of latent states in which it lives to a narrow range of life-compatible possibilities, counteracting the natural tendency for disorder (Friston, 2012)—hence the relationship with thermodynamics. If these states are defined by the organism's phenotype, from the point of view of its internal model they are exactly the states that it expects to be less surprising. Thus, while perceptual inference tries to optimize the belief about hidden causes to explain away sensations, if on the other hand the assumptions defined by the phenotype are considered to be the true causes of the world, interacting with the external environment means that the agent will try to sample those sensations that make the assumptions true, fulfilling its needs and beliefs. *Active inference* becomes a self-fulfilling prophecy. In this view, there is no difference between a desire and a belief: we simply seek the states in which we expect to find ourselves (Friston et al., 2010; Buckley et al., 2017).

For achieving a goal-directed behavior, it is then sufficient to minimize the free energy also with respect to the action (see Equation 7):


(15) $$\begin{array}{l} a=\underset{a}{\text{arg min }}F\left(\mu ,s\right) \end{array}$$

Given that motor control signals only depend on sensory information, we obtain:


(16) $$\begin{array}{l} \overset{\cdot }{a}=-\partial_{a}F\left(\tilde{s},\tilde{\mu }\right)=-\frac{\partial F}{\partial \tilde{s}}\frac{\partial \tilde{s}}{\partial a}=-{\frac{\partial \tilde{s}}{\partial a}}^{T}{\tilde{\Pi }}_{s}{\tilde{\varepsilon }}_{s} \end{array}$$

Minimizing the free energy of all sensory signals is certainly useful, as every likelihood contribution will drive the belief update; however, it requires the knowledge of an inverse mapping from exteroceptive sensations to actions (Baltieri and Buckley, 2019), which is considered a "hard problem" being in general highly non-linear and not univocal (Friston et al., 2010). In a more realistic scenario, only proprioception drives the minimization of free energy with respect to the motor signals; this process is easier to realize since the corresponding sensory prediction is already in the intrinsic domain. Control signals sent from the motor cortex are then not motor commands as in classical views of Optimal Control theories; rather, they consist of predictions that define the desired trajectory. Under this perspective, proprioceptive prediction errors computed locally at the spinal cord serve two purposes that only differ in how these signals are conveyed. They drive the current belief toward sensory observations—happening to realize perception—like for exteroceptive signals. But they also drive sensory observations toward the current belief by suppression in simple reflex arcs that activate the corresponding muscles—thus happening to realize movement (Adams et al., 2013; Parr and Friston, 2018; Versteeg et al., 2021).

In conclusion, perception and action can be seen as two sides of the same coin implementing the common vital goal of minimizing entropy or average surprise. In this view, what we perceive never tries to perfectly match the real state of affairs of the world, but is constantly biased toward our preferred states. This means that action only indirectly fulfills future goals; instead, it continuously tries to fill the gap between sensations and predictions generated from our already biased beliefs.

### 2.5. Variational autoencoders

Variational Autoencoders (VAEs) belong to the family of generative models, since they learn the joint distribution *p*(***z, s***) and can generate synthetic data similar to the input, given a prior distribution *p*(***z***) over the latent space. VAEs use the variational Bayes approach to capture the posterior distribution *p*(***z***|***s***) of the latent representation of the inputs when the computation of the marginal is intractable (Goodfellow et al., 2016). A VAE is composed of two probability distributions, both of which are assumed to be Gaussian: a probabilistic *encoder* corresponding to the recognition distribution *q*(***z***|***s***), and a generative function *p*(***s***|***z***) called probabilistic *decoder* computing a distribution over the input space given a latent representation *z* (**Figure 3C**):


(17) $$\begin{array}{l} \begin{array}{c} q\left(z|s\right)=N\left(z|\mu_{\phi },\Sigma_{\phi }\right)\\ p\left(s|z\right)=N\left(s|\mu_{\theta },\Sigma_{\theta }\right) \end{array} \end{array}$$

Although VAEs have many similarities with traditional autoencoders, they are actually a derivation of the AEVB algorithm when a neural network is used for the recognition distribution (Kingma and Welling, 2014). Unlike other variational techniques, the approximate posterior is generally not assumed to be factorial, but since the calculation of the ELBO gradient $\nabla_{\phi }L_{\theta ,\phi }\left(s\right)$ is biased, a method called *reparametrization trick* is used so that it is independent of the parameters ***ϕ***. This method works by expressing the latent variable ***z*** by a function:


(18) $$\begin{array}{l} z=r\left(\epsilon ,\phi ,s\right) \end{array}$$

Where ***ϵ*** is an auxiliary variable independent of ***ϕ*** and ***s***. The ELBO ${\tilde{L}}_{\theta ,\phi }\left(s\right)$ for a single data point can thus be expressed as:


(19) $$\begin{array}{l} {\tilde{L}}_{\theta ,\phi }\left(s\right)=-D_{KL}\left[q\left(z|s\right)||p\left(z\right)\right]+\frac{1}{M}\overset{M}{\underset{m}{\sum }}\log p\left(s|z_{m}\right) \end{array}$$

Which can be minimized through backpropagation. Here, the KL divergence can be seen as a regularizer, while the second RHS term is an expected negative reconstruction term that depends on all the *m*th components of the latent variable ***z***.

## 3. A framework for flexible intentions

In what follows, we develop a computational theory of the circuitry controlling goal-directed actions in a dynamically changing environment through flexible intentions and discuss its putative neural basis in the PPC and related areas. We first elaborate on intentionality in Active Inference, then provide a proof-of-concept agent endowed with visual input. The theory is exemplified and assessed in the following sections through simulations of visually-guided behaviors. The theoretical work is motivated by basic research showing the critical role of the PPC in goal-directed sensorimotor control through intention coding (Andersen, 1995; Desmurget et al., 1999; Galletti and Fattori, 2018) and extends previous theoretical and applied research on Active Inference (Friston et al., 2009; Pio-Lopez et al., 2016; Lanillos and Cheng, 2018; Limanowski and Friston, 2020) and VAE-based vision support (Rood et al., 2020; Sancaktar et al., 2020). The simulations are inspired by a classical monkey reaching task (Breveglieri et al., 2014).

### 3.1. Flexible intentions

State-of-the-art implementations of continuous Active Inference have proven to successfully tackle a wide range of tasks, from oculomotion dynamics (Adams et al., 2015) to the well-known mountain car problem (Friston et al., 2009). Most simulations involve reaching movements in robotic experiments, where several strategies have been tried for designing goal states, which are expressed in terms of an attractor embedded in the system dynamics. However, there seem to be a few issues regarding biological plausibility. First, the goal state is usually static and the agent is not able to deal with continuously changing environments, expecting that the world will always evolve in the same way (Baioumy et al., 2020). For dynamic goals, one has to use low-level information of sensory signals (e.g., a visual input about a moving target) directly into the high-level dynamics function (Friston, 2011). Second, when goals are specified in an exteroceptive domain, one uses sensory predictions to obtain a belief update direction through backpropagation of the corresponding error (Oliver et al., 2019; Sancaktar et al., 2020). In this case, the same generative model that produces predictions and compares them with the actual observations, has to be duplicated into the system dynamics to further compare the belief with the desired cue. In other words, two specular mechanisms are used for the same model, with additional concerns when the latter can be changed by learning.

A common question seems to be behind these two similar issues: how does dynamic sensory information get available for generating high-level dynamic goals? The same inference process of environmental causes should be at work for the same signal flow, and a goal state should be computed locally without information passed inconsistently. How then to design a flexible exteroceptive attractor that avoids implausible scenarios?

Although the high-level latent state could be as simple as encoding body configurations only, an agent could also maintain a dynamically estimated belief over moving objects in the scene. An intention can then be computed by exploiting this new information to compute a future action goal in terms of body posture, so that the attractor—either defined in the belief domain or at the sensory level—is not fixed but depends on current perceptual and internal representations of the world (but also on past memorized experiences). This intention may also depend on priors generated from higher-level areas (Friston et al., 2011), so that the considered belief is located at an intermediate level between the generative models that produce sensory predictions, and the ones that define its evolution over time. In a non-trivial task, its dynamics may be generally composed of several contributions and not restricted to a single intention: we thus propose to decompose it into a set of functions, each one providing an independent expectation that the agent will find itself in a particular state. The belief is then constantly subject to several forces of two natures: one from lower hierarchical levels—proportional to *sensory prediction errors*—that pulls it toward what the agent is currently perceiving, and one from lateral or higher connections—which we call *intention prediction errors*—that pulls it toward what the agent expects to perceive in the future.

**Figure 1:**
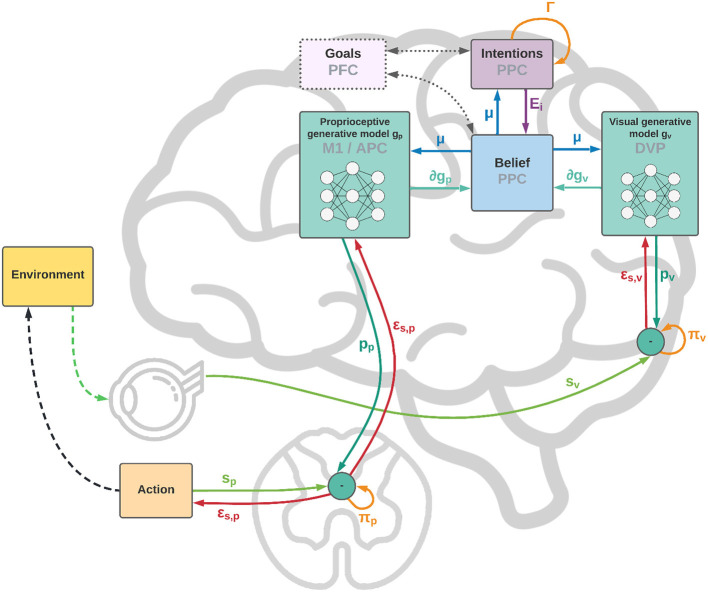
Functional architecture and cortical overlay. The process starts with the computation of future intentions ***h*** (not explicitly represented in the figure) in the PPC under the coordination of frontal and motor areas. In the middle of the sensorimotor hierarchy, the PPC maintains beliefs ***μ*** over the latent causes of sensory observations ***s***_*p*_ and ***s***_*v*_, and computes proprioceptive and visual predictions through the somatosensory and dorsal visual pathways (for simplicity, we have omitted the somatomotor pathway and considered a single mechanism for both motor control and belief inference). The lower layers of the hierarchy compute sensory prediction errors ***ε***_*s*_*p*__ and ***ε***_*s*_*v*__, while the higher layers compute intention prediction errors ***E***_*i*_; both are propagated back toward the PPC, which thus integrates information from multiple sensory modalities and intentions. Free energy is minimized throughout the cortical hierarchy by changing the belief about the causes of the current observation (perception) and by sending proprioceptive predictions from the motor cortex to the reflex arcs (action). An essential element of this process is the computation of gradients ∂***g***_*p*_ and ∂***g***_*v*_ by inverse mappings from the sensations toward the deepest latent states. In this process, intentions act as high-level attractors and the belief propagated down to compute sensorimotor predictions embeds a component directing the body state toward the goals.

As shown in Figure 1, from a neural perspective the PPC is the ideal candidate for a cortical structure computing beliefs over bodily states and flexible intentions: on one hand, being at the apex of the Dorsal Visual Stream (DVS) and other sensory generative models, and on the other linked with motor and frontal areas that produce continuous trajectories and plans of discrete action chunks. The PPC is known to be an associative region that integrates information from multiple sensory modalities and encodes visuomotor transformations—e.g., area V6A is thought to encode object affordances during reaching and grasping tasks (Fattori et al., 2017; Filippini et al., 2017). Moreover, evidence suggests that the PPC encodes multiple goals in parallel during sequences of actions, even when there is a considerable delay between different goals (Baldauf et al., 2008).

In short, the agent constantly maintains plausible hypotheses over the causes of its percepts, either bodily states or objects in exteroceptive domains; by manipulating them, the agent dynamically constructs representations of future states, i.e., intentions, which in turn act as priors over the current belief. Thus, if the job of the sensory pathways is to compute sensory-level predictions, we hypothesize that higher levels of the sensorimotor control hierarchy integrate into the PPC previous states of belief with flexible intentions, each predicting the next plausible belief state.

### 3.2. Dynamic goal-directed behavior in Active Inference

For a more formal definition, we assume that the neural system perceives the environment and receives motor feedback through *J* noisy sensors ***S*** comprising multiple domains (most critically, proprioceptive and visual). Under the VB and Gaussian approximations of the recognition density, we also assume that the nervous system operates on beliefs ***μ*** ∈ ℝ^M^ that define an abstract internal representation of the world. Furthermore, we assume that the agent maintains generalized coordinates up to the 1st order resulting from free energy minimization in the generalized belief space $\tilde{\mu }=\left\{\mu ,\mu^{\prime }\right\}$.

We then define *intentions* ***h***_*k*_ as predictions of target goal states over the current belief ***μ*** computed with the help of *K* functions $i_{k}\left(\mu \right)\in {\mathbb{R}}^{\text{M}}$. Although both belief and intentions could be abstract representations of the world—comprising states in extrinsic and intrinsic coordinates—we assume a simpler scenario in which the intentions operate on beliefs in a common intrinsic motor-related domain, e.g., the joint angles space. As explained before, we assume that there are two conceptually different components in both the belief ***μ*** and the output of the intention functions ***i***_*k*_. The first component could represent the bodily states and serve to drive actions, while the second one could represent the state of other objects—mostly targets to interact with—which can be internally encoded in the joint angles space as well (the reason for this particular encoding will be clear later). These targets could be observed, but they could also be imagined or set by higher-level cognitive control frontal areas such as the PFC or PMd (Genovesio et al., 2012; Stoianov et al., 2016).

For the sake of notational simplicity, we group all intentions into a single matrix ***H*** ∈ ℝ^MxK^:


(20) $$\begin{array}{l} H=i\left(\mu \right)=\left[\begin{array}{ccc} i_{0}\left(\mu \right) & \ldots & i_{K}\left(\mu \right) \end{array}\right]=\left[\begin{array}{ccc} h_{0} & \ldots & h_{K} \end{array}\right] \end{array}$$

Intention prediction errors ***e***_*i*_*k*__ are then defined as the difference between the current belief and every intention:


(21) $$\begin{array}{l} E_{i}=H-\mu =\left[\begin{array}{ccc} e_{i_{0}} & \ldots & e_{i_{K}} \end{array}\right] \end{array}$$

In turn, sensory predictions are produced by a set of generative models ***g***_*j*_, one for each sensory modality. We group the predictions into a prediction matrix ***P***:


(22) $$\begin{array}{l} P=g\left(\mu \right)=\left[\begin{array}{c} g_{0}\left(\mu \right)\\ \vdots \\ g_{J}\left(\mu \right) \end{array}\right]=\left[\begin{array}{c} p_{0}\\ \vdots \\ p_{J} \end{array}\right] \end{array}$$

Note that each term ***p***_*j*_ is a multidimensional sensory-level representation that provides predictions for a particular sensory domain, with its own dimensionality, which we group into a single quantity for notational simplicity. Sensory prediction errors ***ε***_*s*_*j*__ are then computed as the difference between sensations from each domain and the corresponding sensory-level predictions:


(23) $$\begin{array}{l} \varepsilon_{s}=S-P=\left[\begin{array}{c} \varepsilon_{s_{0}}\\ \vdots \\ \varepsilon_{s_{J}} \end{array}\right] \end{array}$$

Under the assumption of independence among intentions and sensations, we can factorize the joint probability of the generative model into a product of distributions for each sensory modality and intention, which expands as follows:


(24) $$\begin{array}{l} p\left(\tilde{\mu },s\right)=p\left(\mu \right)\overset{K}{\underset{k}{\prod }}p\left(\mu_{k}^{\prime }|\mu \right)\cdot \overset{J}{\underset{j}{\prod }}p\left(s_{j}|\mu \right) \end{array}$$

In the following, we will not consider the prior probability over the 0th order belief *p*(***μ***). The other probability distributions are assumed to be Gaussian:


(25) $$\begin{array}{l} \begin{array}{c} p\left(\mu_{k}^{\prime }|\mu \right)=N\left(\mu_{k}^{\prime }|f_{k}\left(\mu \right),\gamma_{k}^{-1}\right)\\ p\left(s_{j}|\mu \right)=N\left(s_{j}|g_{j}\left(\mu \right),\pi_{j}^{-1}\right) \end{array} \end{array}$$

Where ***γ***_*k*_ and ***π***_*j*_ are, respectively, the precisions of intention *k* and sensor *j*. Here, $\mu_{k}^{\prime }$ and ***f***_*k*_ correspond to the *k*th component of the 1st order dynamics function:


(26) $$\begin{array}{l} f_{k}\left(\mu \right)=\lambda e_{i_{k}}+w_{\mu_{k}} \end{array}$$

Where λ is the gain of intention prediction errors ***E***_*i*_. Note that the goal states are embedded into these functions, acting as belief-level attractors for each intention, so that the agent expects to be pulled toward target states with a velocity proportional to the error. Although the generalized belief allows encoding information about the dynamics of the true generative process, in the simple case delineated the agent does not have any such prior. For example, the agent does not know the trajectory of a moving target in advance (whose prior, in a more realistic scenario, would be present and acquired through learning of past experiences) and will update the belief only relying on the incoming sensory information. Nevertheless, the agent maintains (false) expectations about target dynamics, and it is indeed the discrepancy between the evolution of the (real) generative process and that of the (internal and biased) generative model that makes it able to implement a goal-directed behavior.

The prediction errors of the dynamics functions can be grouped into a single matrix:


(27) $$\begin{array}{l} \varepsilon_{\mu }=\mu^{\prime }-\lambda E_{i} \end{array}$$

From Equation (14), we can now compute the free energy derivative with respect to the belief:


(28) $$\begin{array}{l} \overset{\cdot }{\tilde{\mu }}=\left[\begin{array}{c} \overset{\cdot }{\mu }\\ {\overset{\cdot }{\mu }}^{\prime } \end{array}\right]=D\tilde{\mu }-\partial_{\tilde{\mu }}F=\left[\begin{array}{c} \mu^{\prime }+G^{T}\left(\Pi ⊙\varepsilon_{s}\right)+\left(F⊙\varepsilon_{\mu }\right)\Gamma^{T}\\ -\varepsilon_{\mu }\Gamma^{T} \end{array}\right] \end{array}$$

Here, ⊙ is the element-wise product, ***G*** and ***F*** enclose the gradients of all sensory generative models and dynamics functions, while **Π** and **Γ** comprise all sensory and intention precisions:


(29) $$\begin{array}{l} G=\frac{\partial g}{\partial \mu }=\left[\begin{array}{c} \partial g_{0}\\ \vdots \\ \partial g_{J} \end{array}\right]\;\Pi =\left[\begin{array}{c} \pi_{0}\\ \vdots \\ \pi_{J} \end{array}\right]\\ F=\frac{\partial f}{\partial \mu }=\left[\begin{array}{ccc} \partial f_{0} & \ldots & \partial f_{K} \end{array}\right]\;\Gamma =\left[\begin{array}{ccc} \;\gamma_{0} & \ldots & \gamma_{K} \end{array}\right] \end{array}$$

In the following, we will neglect the backward error in the 0th order of Equation (28) since it has a much smaller impact on the overall dynamics, and treat as the actual attractor force the forward error at the 1st order:


(30) $$\begin{array}{l} \overset{\cdot }{\tilde{\mu }}\approx \left[\begin{array}{c} \mu^{\prime }+G^{T}\left(\Pi ⊙\varepsilon_{s}\right)\\ -\varepsilon_{\mu }\Gamma^{T} \end{array}\right]=\left[\begin{array}{c} \mu^{\prime }+\epsilon_{s}\\ \epsilon_{i} \end{array}\right] \end{array}$$

Where ***ϵ***_*s*_ and ***ϵ***_*i*_, respectively, stand for the contributions (in the belief domain) of precision-weighted sensory and intention prediction errors. Considering the 1st order forward error as attractive force instead of the 0th order backward error results in simpler computations since there is no gradient of the dynamics functions to be considered. Further studies are however needed to understand the relationships between these two forces in goal-directed behavior. We can interpret ***γ***_*k*_ as a quantity that determines the relative attractor gain of intention *k*, so that intentions with greater strength have a more significant impact on the overall update direction; these gains could also be modulated by projections from higher-levels areas applying cognitive control. In turn, ***π***_*j*_ corresponds to the confidence about each sensory modality *j*, so that the agent relies more on sensors with higher strength.

Similarly, we can compute control signals by minimizing the free energy with respect to the actions, expressing the mapping from sensations to actions by:


(31) $$\begin{array}{l} \begin{array}{l} \frac{\partial s}{\partial a}=\frac{\partial \mu }{\partial a}\cdot G\\ \;\overset{\cdot }{a}=-\partial_{a}F=-\partial_{a}\mu^{T}\epsilon_{s} \end{array} \end{array}$$

Where ∂_*a*_***μ*** is an inverse model from belief to actions. If motor signals are defined in terms of joint velocities, we can decompose and approximate the inverse model as follows:


(32) $$\begin{array}{l} \partial_{a}\mu =\frac{\partial \theta }{\partial a}\cdot \frac{\partial \mu }{\partial \theta }=\frac{\partial g_{p}}{\partial a}\cdot \frac{\partial \mu }{\partial g_{p}}=\Delta_{t}G_{p}^{-1} \end{array}$$

Where ***θ*** are the joint angles, the subscript *p* indicates the proprioceptive contribution and we approximated ∂_*a*_***g***_*p*_ by a time constant Δ_*t*_ (Oliver et al., 2019). If we assume that the belief over hidden states is encoded in joint angles, the computation of the inverse model may be as simple as finding the pseudoinverse of a matrix. However, if the belief is specified in a more generic reference frame and the proprioceptive generative model is a non-linear function, it could be harder to compute the corresponding gradient, causing additional control problems like temporal delays on sensory signals (Friston, 2011). Alternatively, we can consider a motor control driven only by proprioceptive predictions, so that the control signal is already in the correct domain and may be achieved through simple reflex arc pathways (Adams et al., 2013; Versteeg et al., 2021). In this case, all that is needed is a mapping from proprioceptive predictions to actions:


(33) $$\begin{array}{l} \overset{\cdot }{a}=-\partial_{a}F_{p}=-\Delta_{t}\cdot \pi_{p}\varepsilon_{p} \end{array}$$

Expressing in Equation (31) the mapping from sensations to actions by the product of the inverse model ∂_*a*_***μ*** and the gradient of the generative models allows the control signals to be defined in terms of the weighted sensory contribution ***ϵ***_*s*_, already computed during the inference process. Such an approach may have some computational advantages (as will be explained later), but it is unlikely to be implemented in the nervous system as control signals are supposed to convey predictions and not prediction errors (Adams et al., 2013).

Algorithm 1 outlines a schematic description of the flow of dynamic computations. For simplicity, we used the term "intention" also when describing the dynamics functions and their precisions, but one has to keep in mind the difference between the intention prediction errors ***E***_*i*_, which directly encode the direction toward target states, and the dynamics prediction errors ${ℰ}_{\mu }$, which arise from the derivation of the corresponding probability distributions.

**Algorithm 1:**
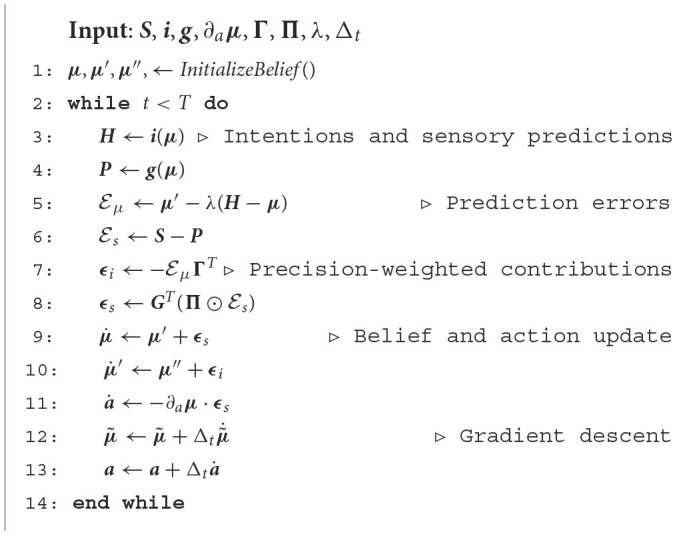
Active Inference agent with flexible intentions.

### 3.3. Neural implementation

**Figure 2:**
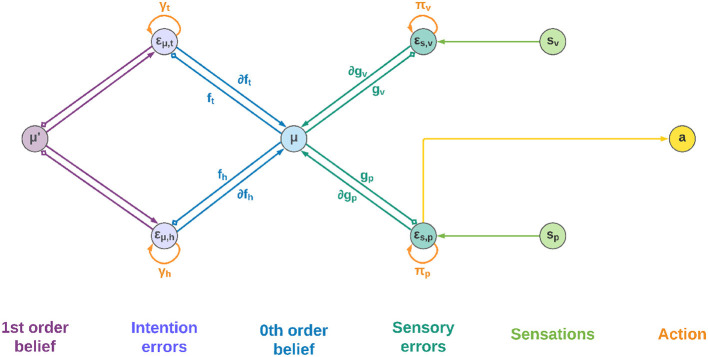
Neuronal representation with two intentions. Small squares stand for inhibitory connections.

Figure 2 shows a schematic neuronal representation of the proposed agent, which further extends earlier perceptual inference schemes (Bogacz, 2017) to full-blown Active Inference. In this simple model, the intentions consist of a single layer with two neurons, and the goal states are implicitly defined in the dynamics functions; however, in a realistic setting the latter would be composed of networks of neurons where these states are explicitly encoded, and non-linear functions could also be used to achieve more advanced behaviors. Note also that intentions ***h***_*k*_ and sensory generative models ***g***_*j*_ are all part of the same architecture, the only difference being the location in the cortical hierarchy.

Low-level prediction errors for each sensory modality are represented by neurons whose dynamics depends on both observations and predictions of the sensory generative models:


(34) $$\begin{array}{l} {\overset{\cdot }{\varepsilon }}_{s_{j}}=s_{j}-g_{j}\left(\mu \right)-\frac{\varepsilon_{s_{j}}}{\pi_{j}} \end{array}$$

Upon convergence of the neural activity, that is, ${\overset{\cdot }{\varepsilon }}_{s_{j}}=0$, we obtain the prediction error computation derived above. In turn, the internal activity of neurons corresponding to high-level prediction errors is obtained by subtracting the generated dynamics function from the 1st order belief:


(35) $$\begin{array}{l} {\overset{\cdot }{\varepsilon }}_{\mu_{k}}=\mu^{\prime }-f_{k}\left(\mu \right)-\frac{\varepsilon_{\mu_{k}}}{\gamma_{k}} \end{array}$$

Having received information coming from the top and bottom of the hierarchy, the belief is updated by integrating every signal:


(36) $$\begin{array}{l} \overset{\cdot }{\mu }=\overset{J}{\underset{j}{\sum }}\partial g_{j}\varepsilon_{s_{j}}+\overset{K}{\underset{k}{\sum }}\partial f_{k}\varepsilon_{\mu_{k}} \end{array}$$

Which parallels the update formula derived above (Equation 28). Correspondingly, the 1st order component of the belief is updated as follows:


(37) $$\begin{array}{l} {\overset{\cdot }{\mu }}^{\prime }=-\overset{K}{\underset{k}{\sum }}\varepsilon_{\mu_{k}} \end{array}$$

The belief is thus constantly pushed toward a direction that matches sensations on one side and intentions on the other. We adopted the idea that the slow-varying precisions are encoded as synaptic strengths (Bogacz, 2017), but alternative views consider them as gains of superficial pyramidal neurons (Bastos et al., 2012). In any case, they could be dynamically optimized during inference in a direction that minimizes free energy—e.g., if a sensory modality does not help predict sensations, its weight will decrease. This is also true for the intention weights: by dynamically changing during the movement, they can act as modulatory signals that select the best intention to realize at every moment, which can be useful for solving simultaneous or sequential tasks. Nonetheless, the distinction is purely conceptual as the agent does not discriminate between modulating a future intention or increasing the confidence of a sensory signal. At the belief level, every element just follows the rules of free energy minimization.

## 4. Method

**Figure 3:**
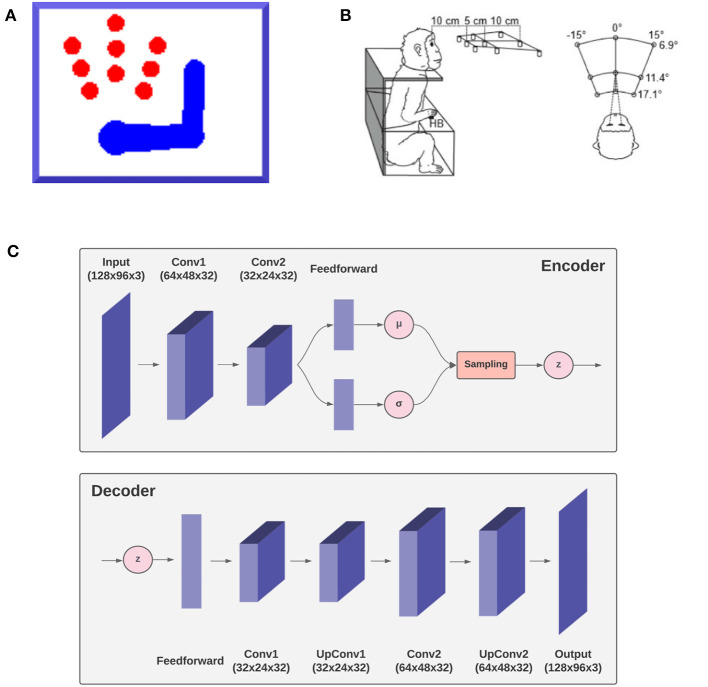
Simulation outline. The agent, a simulated 3-DoF actuated upper limb shown in **(A)** is set to reach one of the nine red circle targets as in the reference monkey experiment (Breveglieri et al., 2014) outlined in **(B)**. The agent is equipped with a fixed virtual camera providing peripersonal visual input and a visual model, the decoder ***g***_*v*_ of a VAE shown in **(C)** simulating functions of the DVS.

To demonstrate the feasibility of the approach and its capacity to successfully implement goal-directed behavior in dynamic environments, we simulated an agent consisting of an actuated upper limb with visual and proprioceptive sensors that allow it to perceive and reach static and moving targets within its reach.^1^ Figure 3A shows the size and position of the targets, as well as limb size and a sample posture. Since the focus here was on theoretical aspects, we simulated just a coarse 3-DoF limb model moving on a 2D plane. However, the approach easily generalizes to a more elaborated limb model and 3D movements. In the following, we describe the agent, the specific implementation, and the simulated task. Then, in the Results section we assess the agent's perceptual and motor control capabilities in static and dynamic conditions. The static condition simulated a typical monkey reaching task of peripersonal targets as in Figure 3 (Breveglieri et al., 2014). In turn, the dynamic condition involved a moving target that the agent had to track continuously.

### 4.1. Delayed reaching task

The primary testbed task is a simplified version of a delayed reaching monkey task in which a static target must be reached with a movement that can only start after a delay period (Breveglieri et al., 2014). Delayed actions are used to separately investigate neural processes related to action preparation (e.g., perception and planning) and execution in goal-directed behavior, and are thus useful to analyze the two main computational components of free energy minimization, namely, perceptual and active inference, which otherwise work in parallel. Delayed reaching could be implemented using various approaches: the update of the posture component of the belief dynamics could be blocked by setting the intention gain λ to zero during inference (implemented here): in this way, there are no active intentions and the belief only follows sensory information. Alternatively, action execution could be temporarily suspended by setting to zero the proprioceptive precision, so that the agent still produces proprioceptive predictions but does not trust their prediction errors: in this scenario, the belief dynamics includes a small component directed toward the intention, but the discrepancy produced is not minimized through movement.

Reach trials start with the hand placed on a home button (HB) located in front of the body center (i.e., the “neck”), and the belief is initialized with this configuration. Then one of the 9 possible targets of the reference experiment (Figure 3) is lit red. Follows a delay period of 100 time steps during which the agent is only allowed to perceive the visible target and the limb, and the inference process can only change the belief. After that, the limb is allowed to move and the joint angles are updated according to Equation (38). As in the reference task, upon target reaching the agent stops for a sufficiently long period, i.e., a total of 300 time steps per trial. After that, the agent reaches back the HB (not analyzed here). The simulation included 100 repetitions per target, i.e., 900 trials in total.

### 4.2. Body

The body consists of a simulated monkey upper limb composed of a moving torso attached to an anchored neck, an upper arm, and a lower arm, as shown in Figure 3. The three moving segments are schematized as rectangles, each with unit mass, while the joints (shoulder, elbow) and the tips (neck, hand) as circles. The proportions of the limb segment and the operating range of the joint angles were derived from monkey data *Macaca mulatta* (Kikuchi and Hamada, 2009). The state of the limb and its dynamics are described by the joint angles ***θ*** and their first moment $\overset{\cdot }{\theta }$. We assume noisy velocity-level motor control, whereby the motor efferents *a* noisily control the first moment of joint angles with zero-centered Gaussian noise:


(38) $$\begin{array}{l} \overset{\cdot }{\theta }=a+w_{a} \end{array}$$

### 4.3. Sensors

The agent receives information about its proprioceptive state and visual context. Simplified peripersonal visual input ***s***_*v*_ was provided by a virtual camera that included three 2D color planes, each of them 128 x 96 pixels in size. The location and orientation of the camera were fixed so that the input provided full vision of peripersonal targets and the entire limb in any possible limb state within its operating range. The limb could occlude the target in some configurations.

As in the simulated limb, the motor control system also receives proprioceptive feedback through sensors ***s***_*p*_, providing noisy information on the true state of the limb (Tuthill and Azim, 2018; Versteeg et al., 2021). We further assumed that ***s***_*p*_ provides a noisy reading of the state of all joints only in terms of joint angles, ignoring other proprioceptive signals such as force and stretch (Srinivasan et al., 2021), which the Active Inference framework can natively incorporate.

### 4.4. Belief

We assume that both the orders of the generalized belief $\tilde{\mu }$ comprise three components: (i) beliefs ${\tilde{\mu }}_{a}$ over arm joint angles, or posture; (ii) beliefs ${\tilde{\mu }}_{t}$ over the target location represented again in the joint angles space—i.e., the posture corresponding to the arm touching the target; and (iii) beliefs ${\tilde{\mu }}_{h}$ over a memorized HB configuration. Thus, ***μ*** = [***μ***_*a*_, ***μ***_*t*_, ***μ***_*h*_]. Note that the last two components can be interpreted as *affordances*, allowing the agent to implement interactions in terms of bodily configurations (Pezzulo and Cisek, 2016).

### 4.5. Sensory model

The sensory generative distribution has two components, one for each sensory modality: a simplified proprioceptive model ***g***_*p*_(***μ***) and a full-blown visual model ***g***_*v*_(***μ***):


(39) $$\begin{array}{l} g\left(\mu \right)=\left[\begin{array}{c} g_{p}\left(\mu \right)\\ g_{v}\left(\mu \right) \end{array}\right] \end{array}$$

Since the belief is already in the joint angles domain, we implemented a simple proprioceptive generative model ***g***_*p*_(***μ***) = ***G***_*p*_***μ*** = ***μ***_*a*_, where ***G***_*p*_ is a mapping that only extracts the first component of the belief:


(40) $$\begin{array}{l} G_{p}=\left[\begin{array}{ccc} 𝕀 & 0 & 0 \end{array}\right] \end{array}$$

Where **0** and **𝕀** are respectively 3 x 3 zero and identity matrices. Note that ***g***_*p*_(***μ***) could be easily extended to a more complex proprioceptive mapping if the body and/or joint sensors have a more complex structure and the belief has a richer and abstract representation.

In turn, the visual generative model ***g***_*v*_ is the decoder component of a VAE (see Figure 3C). It consists of one feedforward layer, two transposed convolutional layers, and two standard convolutional layers needed to smooth the output. Its latent space is composed of two elements, representing the joint angles of arm and target (example in **Figure 13**). The first component is used to generate, in the visual output, an arm with a specific joint configuration, while the second component is used to produce only the image of the target through direct kinematics of every joint angle. The VAE was trained in a supervised manner for 100 epochs on a dataset comprising 20.000 randomly drawn body-target configurations that uniformly spanned the entire operational space, and the corresponding visual images. The target size varied with a radius ranging from 5 to 12 pixels.

The proprioceptive gradient ∂***g***_*p*_ simplifies to the mapping ***G***_*p*_ itself, while the visual component ∂***g***_*v*_ is the gradient of the decoder computed by backpropagation. Since the Cartesian position of the target is encoded in joint angles, this gradient implicitly performs a kinematic inversion. Therefore, predictions ***P*** and prediction errors ${ℰ}_{s}$ take the form:


(41) $$\begin{array}{l} P=\left[\begin{array}{c} \mu_{a}\\ g_{v}(\mu ) \end{array}\right]\;{ℰ}_{s}\text{ =}\left[\begin{array}{c} s_{p}-\;\mu_{a}\\ s_{v}-\;g_{v}(\mu ) \end{array}\right] \end{array}$$

Note that defining sensory predictions on both proprioceptive and visual sensory domains allows the agent to perform efficient goal-directed behavior also in conditions of visual uncertainty, e.g., due to low visibility. Indeed, since the belief is maintained over time, the agent remembers the last known target position and can thus accomplish reaching tasks also in case of temporarily occluded targets.

### 4.6. Intentions

**Figure 4:**
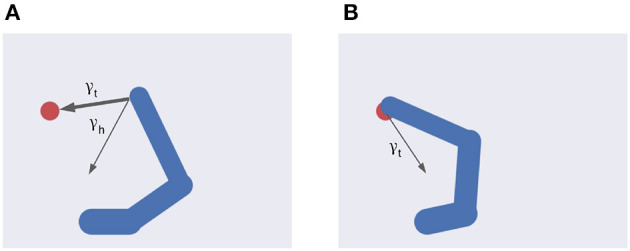
Multiple intentions. Intention prediction errors of variable strength (arrow width) controlled by intention precisions point at different target states. **(A)** A stronger target attraction (the red circle) implements the reaching action. **(B)** A stronger attraction of the invisible but previously memorized HB implements the return-to-home action.

Stepping on the proposed formalization (Equation 20), we define two specific intentions (Figure 4) as follows:


(42) $$\begin{array}{l} H=\left[\begin{array}{cc} i_{t}\left(\mu \right) & i_{h}\left(\mu \right) \end{array}\right]=\left[\begin{array}{cc} I_{t}\mu & I_{h}\mu \end{array}\right]=\left[\begin{array}{cc} \mu_{t} & \mu_{h}\\ \mu_{t} & \mu_{t}\\ \mu_{h} & \mu_{h} \end{array}\right] \end{array}$$

Here ***h***_*t*_ = ***i***_*t*_(***μ***) defines the agent's expectation that the arm belief is equal to the joint configuration corresponding to the target to be reached, and it is implemented as a simple mapping ***I***_*t*_ that sets the first belief component equal to the second one. In turn, the intention ***h***_*h*_ = ***i***_*h*_(***μ***) encodes the future belief of the agent that the arm will be at the HB position. The two intention mappings are defined by:



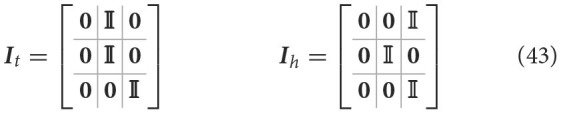



The corresponding intention prediction errors are then:


(44) $$\begin{array}{l} E_{i}=\left[\begin{array}{cc} e_{i_{t}} & e_{i_{h}} \end{array}\right]=\left[\begin{array}{cc} h_{t}-\mu & h_{h}-\mu \end{array}\right]=\left[\begin{array}{cc} \mu_{t}-\mu_{a} & \mu_{h}-\mu_{a}\\ 0 & 0\\ 0 & 0 \end{array}\right] \end{array}$$

These errors provide an update direction respectively toward the target and HB joint angles. As there is no intention to move the target or the HB, the second and third components of the prediction errors will be zero.

### 4.7. Precisions

Free energy minimization and Predictive Coding in general heavily depend on precisions modulation. To investigate their role, we parameterized the relative precisions of each intention and sensory domain with parameters α and β as follows:


(45) $$\begin{array}{llll} \Pi & =\left[\begin{array}{c} \pi_{p}\\ \pi_{v} \end{array}\right]=\left[\begin{array}{c} 1-\alpha \\ \alpha \end{array}\right] & \Gamma & \text{= [}\begin{array}{cc} \gamma_{t} & \gamma_{h} \end{array}\text{] = [}\begin{array}{cc} 1-\beta & \beta \end{array}\text{]} \end{array}$$

The parameter α controls the relative strength of the error update due to proprioception and vision, while the parameter β controls the relative attraction by each intention. With these parameters, the sensory and intention weighted contributions are unpacked as follows:


(46) $$\begin{array}{ll} \epsilon_{s} & =\left(1-\alpha \right)\cdot G_{p}^{T}\varepsilon_{s_{p}}+\alpha \cdot \partial g_{v}^{T}\varepsilon_{s_{v}} \end{array}$$

(47) $$\begin{array}{ll} \epsilon_{i} & =-\mu^{\prime }+\lambda \left[\left(1-\beta \right)\cdot e_{i_{t}}+\beta \cdot e_{i_{h}}\right] \end{array}$$

Equation (46) shows the balance between visual and proprioceptive information. For example, if α = 0 the agent will only use proprioceptive feedback, while for α = 1 the belief will be updated only relying on visual feedback. Note that these are extreme conditions—e.g., the former may correspond to null visibility—and typical sensory systems provide balanced feedback. In turn, Equation (47) spells out the control of belief attraction. The agent will follow the first intention when β = 0, or the second one when β = 1 (Figure 4). Note that the introduction of a possible competing reach movement creates a conflict among intentions aiming to fulfill opposing goals (e.g., for intermediate values of β) while the agent can physically realize only one of them at a time (Figure 4). Thus, we assume that the control of intention selection is realized through mutual inhibition and higher-level bias. Finally, the parameter λ controls the overall attractor magnitude (see also Equation 26).

We can also use the precision parameter α to manipulate the strength of the free energy derivative with respect to the actions as follows:


(48) $$\begin{array}{l} \overset{\cdot }{a}=-\Delta_{t}\left(1-\alpha \right)\cdot \varepsilon_{s_{p}} \end{array}$$

Note that by increasing α—i.e., more reliability on vision—the magnitude of the belief update remains constant, while action updates decrease because the agent becomes less confident about its proprioceptive information. Also, one could differentially investigate the effect of precision strength on belief and action by directly manipulating the precisions—e.g., visual precision π_*v*_ may include different components that follow the belief structure:


(49) $$\begin{array}{l} \pi_{v}=\left[\alpha ,\pi_{v_{t}},\pi_{v_{h}}\right] \end{array}$$

Where we used the parameter α only for the arm belief. For example, when α = 0 and π_*v*_*t*__ > 0, the target belief is updated using visual input while the arm moves only using proprioception, a scenario that emulates movement in darkness with a lit target.

## 5. Results

In the following, we assess the capacities of the intention-driven Active Inference agent to perceive and perform goal-directed actions in reaching tasks with static and dynamic visual targets. The main testbed task was delayed reaching, but we simulated several other conditions.

Sensorimotor control that implements goal-directed behavior was investigated in various sensory feedback conditions, including pure proprioceptive or mixed visual and proprioceptive, in which the VAE decoder provided support for dynamic estimation of visual targets and bodily states. The latter is the typical condition of performing reaching actions and allows greater accuracy (Keele and Posner, 1968). In an additional *baseline* (BL) condition, the target was estimated by the decoder, but the movement was performed without visual feedback or proprioceptive noise, to allow comparisons with the typical approach in previous continuous Active Inference studies, e.g., Pio-Lopez et al. (2016). We also investigated the effects of sensory and intention precisions, motor control type, and movement onset policy. Finally, we analyzed the visual model and the nature of its gradients to provide critical information about the causes of the observed motor behavior.

Action performance was assessed with the help of several measures: (i) *reach accuracy*: success in approaching the target within 10 pixels of its center, i.e., the hand touching the target; (ii) *reach error*: *L*^2^ hand-target distance at the end of the trial; (iii) *reach stability*: standard deviation of *L*^2^ hand-target distance during the period from target reach to the end of the trial, in successful trials; (iv) *reach time*: number of time steps needed to reach the target in successful trials. We also assessed target perception through analog measures based on the *L*^2^ distance between the target location and its estimate transformed from joint angles into visual position by applying the geometric (forward) model. Specifically, we defined the following measures: (v) *perception accuracy*: success in estimating the target location within 10 pixels; (vi) *perception error*: *L*^2^ distance between the true and estimated target position at the end of the trial; (vii) *perception stability*: standard deviation of the *L*^2^ distance between the target position and its estimation during the period starting from successful estimation until end of the trial; (viii) *perception time*: number of time steps needed to successfully estimate the target position.

**Figure 5:**
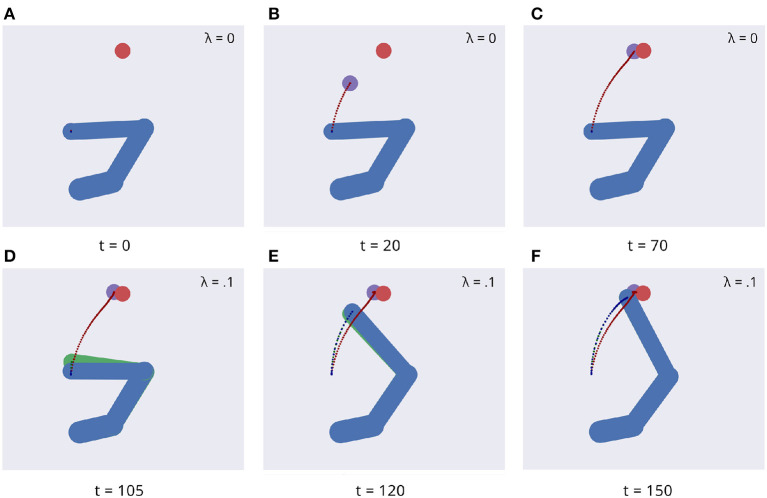
Dynamics of the delayed reaching task. At trial onset **(A)** a visual target (red circle) appears, the arm (in blue) is located on the HB position, and the arm belief (in green) is set at the true arm state. During the delay period, the perceptual inference process gradually drives the target belief (purple circle) toward the real position **(B, C)**. During this phase, the intention gain λ is set to 0, so that movement is inhibited and the arm belief does not change given the unchanged proprioceptive evidence. After movement onset, the arm freely follows its belief **(D, E)** until they both arrive at the goal state **(F)**.

**Figure 6:**
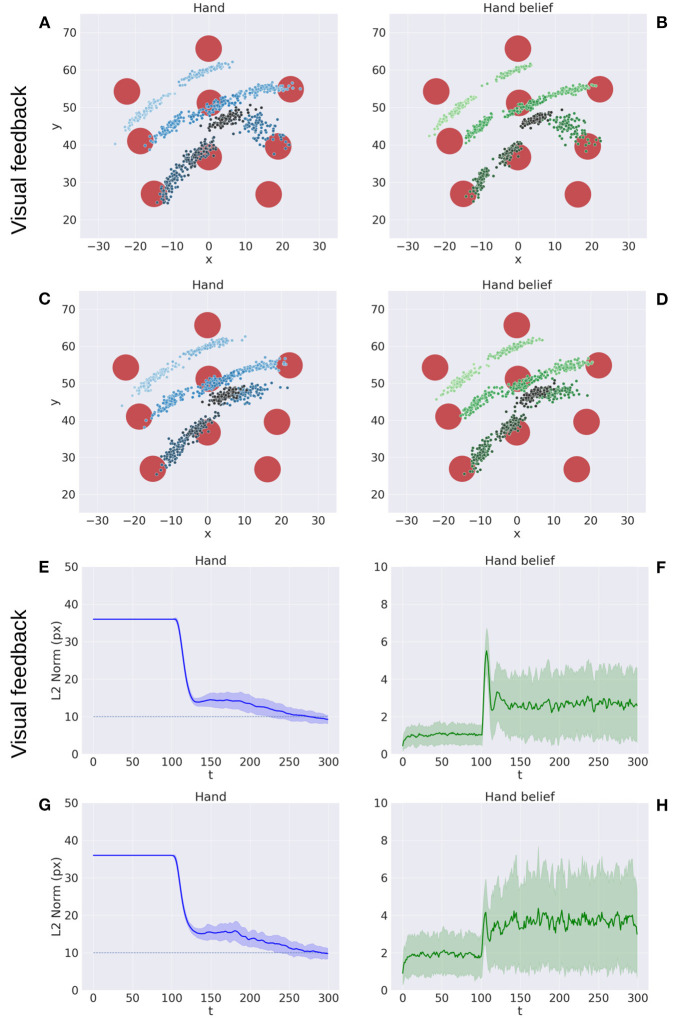
Performance of the delayed reaching task. **(A–D)** Spatial distribution of hand positions **(A, C)** and corresponding beliefs **(B, D)** per target at the end of the reach movements, with **(A, B)** and without **(C, D)** visual feedback. Each point represents a trial (100 trials per target). Reach error **(E, G)** and belief error **(F, H)** over time, with **(E, F)** and without **(G, H)** visual feedback (bands represent C.I.). The reach criterion of the hand-target distance is visualized as a dotted line. *L*^2^ norm for the hand belief is computed by the difference between real and estimated hand positions. Reaching with visual feedback resulted in a more stable hand belief.

Figure 5 illustrates key points of the delayed reaching task. During the delay period (Figures 5A–C), the posture does not change since the joint angles only follow the arm belief, which is kept fixed, while the target belief is attracted by the sensory evidence and gradually shifts toward it. When movement is allowed (Figures 5D–F) by setting λ > 0 and β = 0.1, the combined attractor produces a force that moves the arm belief toward the target, generating proprioceptive predictions—therefore motor commands—that let the real arm follow this trajectory. Reaching performance is summarized in Figure 6. Panels A-D show spatial statistics of the final hand location (with the corresponding belief) for each target, separately for reaching with proprioception only or proprioceptive and visual sensory feedback. Descriptive statistics revealed an important benefit of visual feedback (Figures 6E–H), in parallel with classical behavioral observations (Keele and Posner, 1968): reach accuracy was higher (with: 88.28%; without: 83.72%) and both reach stability and arm belief error were considerably better with visual feedback as well (stability: 1.35; error: 1.98px) compared to the condition with only proprioception (stability: 1.78; error: 2.87px).

### 5.1. Precision balance

**Figure 7:**
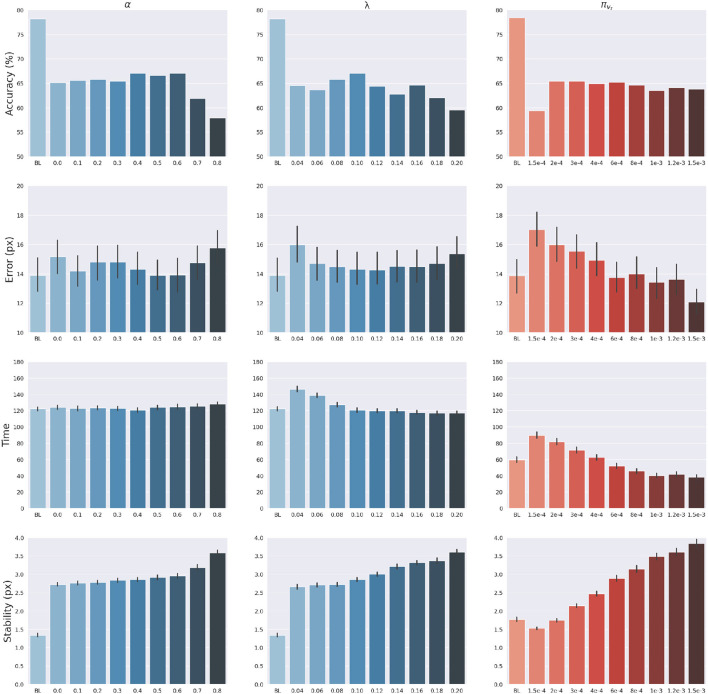
Effects of precision balance. Reach accuracy (1st row), reach error, i.e., *L*^2^ hand-target distance (2nd row), reach time (3rd row), and reach stability (4th row). Reach performance is shown as a function of arm sensory precision α **(left)** and attractor strength λ **(middle)**. In turn, perception performance is shown as a function of target sensory precision π_*v*_*t*__ **(right)**. Vertical bars represent C.I.

The effects of sensory feedback led to a further systematic assessment of the effects of sensory and intention precisions α, π_*v*_*t*__ and λ (see Equations 45, 49). The assessment was carried out following the structure of the delayed reaching task. We varied the above precisions one at a time, using levels shown on the abscissas in Figure 7, while keeping the non-varied precisions at their default values. Note that α = 0 corresponds to reaching without visual feedback, while the conditions α>0 may be interpreted as reaching with different levels of arm visibility. We recall that the baseline condition (BL) performs reaching movements without visual feedback and proprioceptive noise, i.e., α = 0 and *w*_*p*_ = 0. To obtain a systematic evaluation, each condition was run on a rich set of 1,000 randomly selected targets that covered the entire operational space. Finally, we only considered the target-reaching intention, i.e., β = 0; everything else was the same as in the main task.

**Figure 8:**
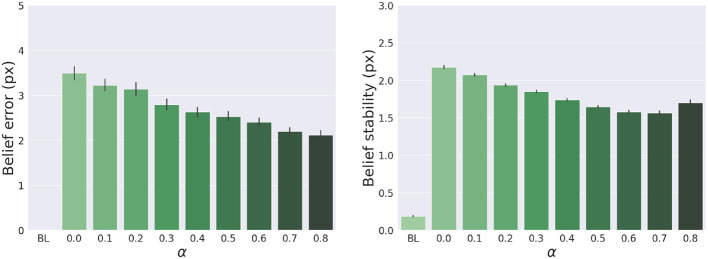
Belief error and stability—representing the difference between real and estimated hand positions—for different values of α. Vertical bars represent C.I.

The results are shown in Figure 7. The panels in the left column show the effect of α compared to the BL agent with noiseless proprioception. Active Inference with only proprioception (i.e., α = 0) has a lower reach accuracy and higher error, while the best performance is obtained with balanced proprioceptive and visual input, in corroboration with the observations of the basic delayed reaching task. In the latter case, the motor control circuitry continuously integrates all available sensory sources to implement visually-guided behavior (Saunders and Knill, 2003). However, accuracy and stability rapidly decrease for excessively high values of α, due to the discrepancy in update directions between the belief—which makes use of all available sensory information, including the more precise visual feedback—and action—which in this case relies on excessively noisy proprioception. Furthermore, as in the main experiment, the effects of visual precision are evident in the stability of the arm belief, which gradually improves with increasing values (Figure 8): In addition to the reliability of the visual input, this effect is also a consequence of the smaller action updates due to the reduced proprioceptive precision.

In turn, the panels in the middle column of Figure 7 reveal the effects of the attractor gain λ; to remind the reader, the greater the gain, the greater the contribution of intention prediction errors in the belief updates. The results show that as the intention gain λ increases reach accuracy generally improves, and the number of time steps needed to reach the target decreases. However, beyond a certain level, the accuracy tends to decrease since the trajectory dynamics becomes unstable; thus, excessively strong action drag is counterproductive to the implementation of smooth movements. Finally, the panels in the right column of Figure 7 show the effects of the target precision π_*v*_*t*__, which directly affects the quality of target perception. Note that better performances are generally obtained in terms of accuracy, error, and perception time for values of π_*v*_*t*__ higher than the arm visual precision, which corresponds to a classical effect of contrast on perception, but also means here that the arm and target beliefs follow different dynamics.

### 5.2. Motor control

We described earlier two different ways of implementing motor control in Active Inference: making use of all sensory information, or proprioception only. The first method requires significantly more computations since the agent needs to know the inverse mapping from every sensory domain to compute the control signals. However, given the assumptions we made, this approach is potentially more stable because it updates both belief and action with the same information. On the other hand, a pure proprioception-based control mechanism could produce potentially incorrect movements because the motor control commands result from comparing proprioceptive predictions with noisy observations. Greater cost-effectiveness of the second method thus might come at the cost of worsened performances, which we investigate here.

**Figure 9:**
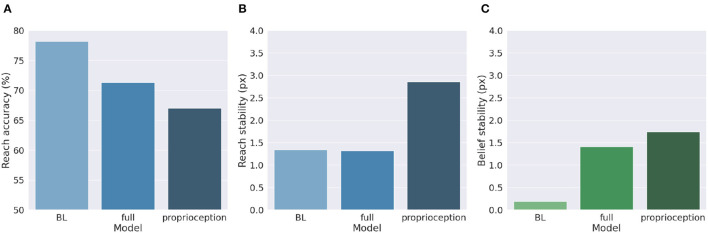
Motor control methods. Reach accuracy **(A)**, reach stability **(B)**, and belief stability **(C)** for a BL agent and the two different implementations of motor control, based either on all sensory information (full control) or on proprioception only.

Figure 9 shows a comparison of the two control methods and the BL agent, evaluated under the same conditions we used to investigate precision balance, including 1,000 random targets. Performance was measured in terms of belief and reach stability and reach accuracy. The results reveal, first, that the expected decreased belief stability of the full model with respect to the BL agent (Figure 9C) does not affect hand stability (Figure 9B), although the proprioceptive noise apparently contributed to decreased reach accuracy (Figure 9A). More importantly, the results confirm our expectations that pure proprioception control has considerably lower reach stability caused by incorrect update directions of the motor control signals, resulting in a greater decreased reach accuracy relative to the full model.

### 5.3. Movement onset policy

We also investigated the effects of movement onset using several policies, which differ by the duration of the period of pure perception preceding full Active Inference. One such policy we investigate here is characteristic of actions performed under time pressure, in which movement starts along with perception, i.e., action is *immediate*. Another policy that could be considered typical for acting under normal conditions has movement beginning with the satisfaction of a certain perception criterion. This policy *dynamically* deliberates the onset of movement. Various perception criteria could be used: here, the action starts when the norm of the target belief $\overset{\cdot }{\mu_{t}}$ remained below a given threshold (i.e., 0.01) for a certain period (i.e., 5 time steps). These parameters were arbitrarily chosen in consideration of exploratory delayed reaching simulations. Finally, we include the previously used delayed action policy in which movement onset is delayed by a *fixed* period (here, 100 time steps, sufficient to obtain a precise target estimation). To obtain systematic observations, each policy was again run on 1,000 randomly selected targets. Measurements included reach and perception accuracy, motor control stability after reach, target perception stability, as well as reach time since the beginning of the trial or after movement onset.

**Figure 10:**
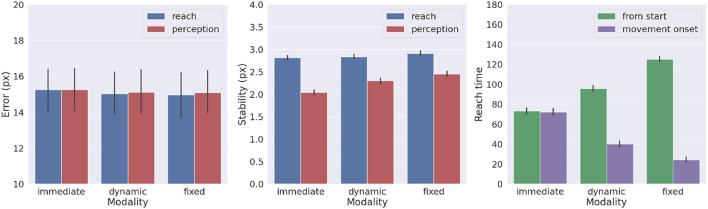
Effects of movement onset policy. Reach error **(left)**, stability **(middle)**, and time **(right)** across several policies (immediate, dynamic, and fixed delay). Vertical bars represent C.I.

Figure 10 shows the results with the three different policies. Although the reach error is approximately the same in all tested conditions, the agent controlled by the immediate policy reached the target within the lowest total number of time steps: target perception and intention setting were dynamically computed along with movement onset. However, if we consider the total task time, the number of time steps is the highest in this condition, since the arm belief and the arm itself move along with the slow visual target estimation. In turn, if the agent starts the movement when the uncertainty about the target position is already minimized (either in the *dynamic* or *fixed* condition), the movement time decreases, although if added on top of the perception time results in slower actions relative to the immediate movement condition. Finally, we note that target perceptual stability somewhat decreases for dynamic and fixed policies; this somewhat unexpected result is encouraging for dynamic target tracking tasks in which immediate movement onset is mandatory.

### 5.4. Tracking dynamic targets

In a second testbed task, the agent was required to track a smooth-moving target whose initial location was randomly chosen from the entire operational space. In each trial, the targets received an initial velocity of 0.1px per step in a direction uniformly spanning the 0–360° range. When the target reached a border, its movement was reflected. As in the previous simulations, the belief was initialized at the HB configuration and the trial time limit was 300 time steps. However, for the agent to correctly follow the targets, both the belief and action were dynamically and continuously inferred in parallel, i.e., without a pure perceptual period.

**Figure 11:**
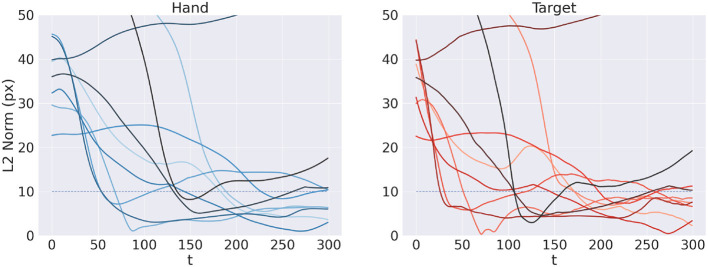
Tracking dynamic targets. Reach **(left)** and perception **(right)** error over time for 10 random trials.

Figure 11 shows the reach trajectory in dynamic target tracking for 10 random trials. The left panel shows the evolution over time of *L*^2^ hand-target distance, while the right panel represents the error between estimated and true target positions. The results suggest that the agent is generally able to correctly and dynamically estimate the beliefs over both target and arm for almost every trial, also in the case of moving targets. In some cases however, mainly when the target is out of reach, it is temporarily or permanently "lost" in terms of its belief, which has also the consequences of losing the target in terms of reach. Further analysis with a more realistic bodily configuration and visual sensory system—as well as comparisons with actual kinematic data—should provide further insights into the capabilities of Active Inference to perform dynamic reaching.

### 5.5. Free energy minimization

Here we illustrate the dynamics of free energy minimization in delayed reaching, which is at the heart of continuous Active Inference. To that aim, we run 10 new reaching trials with static and dynamic targets and recorded the free energy derivatives with respect to generalized belief and action.

**Figure 12:**
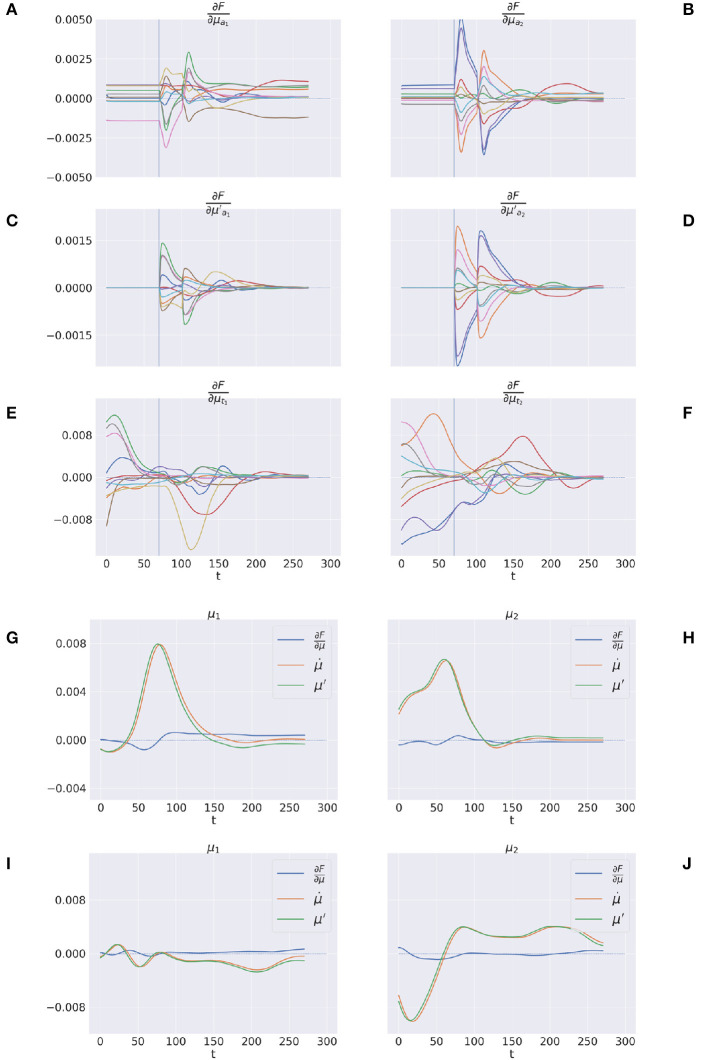
Free energy minimization. **(A–F)** Free energy derivative with respect to the 0th and 1st order belief for arm **(A–D)** and target **(E, F)**. **(G–J)** Comparison between the reference frames of the belief—the path of the mode $\overset{\cdot }{\mu }$ and the mode of the path ***μ***′—for sample static **(G, H)** and dynamic **(I, J)** trials. The left/right columns refer to the arm/forearm segments. Trials data are smoothed with a 30 time-step moving average.

Figures 12A–F shows the trajectory of the free energy derivatives with respect to the arm and target components during delayed reaching of a static target; the two columns show the trends for the last two joints, i.e., the arm and forearm segments, that most strongly articulate the reaching action. Note that the gradients of the free energy with respect to the target belief are rapidly minimized during the initial perceptual phase while the arm gradients remain still. Upon action execution (indicated by a vertical line), the arm gradients rapidly change as well, resulting in updated proprioceptive predictions that drive arm movements. However, arm movements cause changes in the visuals scene, resulting in a secondary effect over the just minimized free energy on target belief. Figures 12G–J goes even deeper, showing a direct comparison between $\overset{\cdot }{\mu }$, ***μ***′, and the difference $\overset{\cdot }{\mu }-\mu^{\prime }$, on sample static (G-H) and dynamic (I-J) targets. We recall that free energy minimization implies that the two reference frames (the path of the mode $\overset{\cdot }{\mu }$ and the mode of the path ***μ***′) should overlap at some point in time, when the agent has inferred the correct trajectory of the generalized hidden states. This is crucial especially in dynamic reaching, in which the aim is to capture the instantaneous trajectory of every object in the scene. The decreasing free energy gradients (blue lines) show that this aim is indeed successfully achieved in both static and dynamic tasks.

### 5.6. Visual model analysis

**Figure 13:**
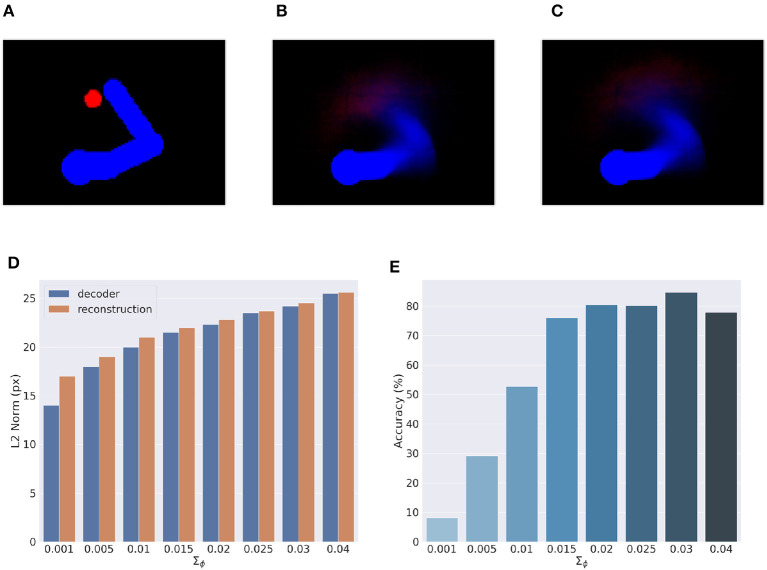
Visual model analysis. **(A–C)** Sample visual observation **(A)** and its decoding from joint angles **(B)** and through a complete encoding-decoding process **(C)**. **(D, E)** Visual model performance. Quality of perception is measured as the *L*^2^ norm between observed and reconstructed images **(D)** and accuracy of Active Inference **(E)**, as a function of the recognition density variance Σ_ϕ_.

Here, we provide an assessment of the visual model whose performance is critical for accurate visually-guided motor control. To recall, the visual model is implemented with a VAE trained offline to reconstruct images of arm-target configurations such as the one in Figure 13A. A critical VAE parameter is the variance of the recognition (encoder) density Σ_ϕ_ (see Equation 17). We therefore evaluated its effect on perception and action by training several VAEs with different variance levels. VAE performance was assessed on other 10.000 randomly selected configurations that uniformly sampled the space, with a target size of 5 pixels (the default condition for the Active Inference tests).

Most critical was the VAE capacity to generate adequate images of joint arm-target configurations, which we measured with the help of the *L*^2^ norm between visual observations, and VAE-generated images. To provide more insights on the two VAE processes, decoding and encoding, we proceeded as follows: first, *decoding* was assessed by generating images for given body-target states such as that in Figure 13B. The decoded images were compared with the ground truth images produced by applying the geometric model for the same state of the body target (Figure 13A). Second, full VAE performance was assessed by computing the average *L*^2^ norm between observed images and their full VAE *reconstruction*, i.e., first encoding and then decoding them (as in Figure 13C). Third, we directly assessed the specific effects of the recognition density variance on Active Inference using the BL condition of the delayed reaching task as a measure.

Figure 13D represents the results of the perceptual assessment tests, showing the *L*^2^ norm between the original and generated images as a function of recognition density variance. As expected, lower variances generally resulted in lower errors with respect to both pure decoding and full encoding-decoding. Surprisingly, however, the accuracy of Active Inference in the reaching task behaved somewhat differently: the best accuracy was obtained not for predictions with low variance, but for intermediate variance levels (Figure 13E). This could be explained by the fact that low-variance images imply highly non-linear gradients that prevent correct gradient descent on free energy. On the other hand, as the variance increases the reconstructed image becomes somewhat blurred, which helps obtain a smoother gradient that correctly drives free energy minimization and therefore improves movement accuracy (more on this in the next section). However, as the variance continues to increase, the reconstructed images become too blurry, degrading both belief inference and motor control.

### 5.7. Visual gradient analysis

To further investigate the cause of the unexpected low variance issue, we analyzed the consistency of the visual gradient ∂***g***_*v*_ of the decoder for several encoder variance values. To this aim, we computed the gradients for different reference states over the entire operational space.

**Figure 14:**
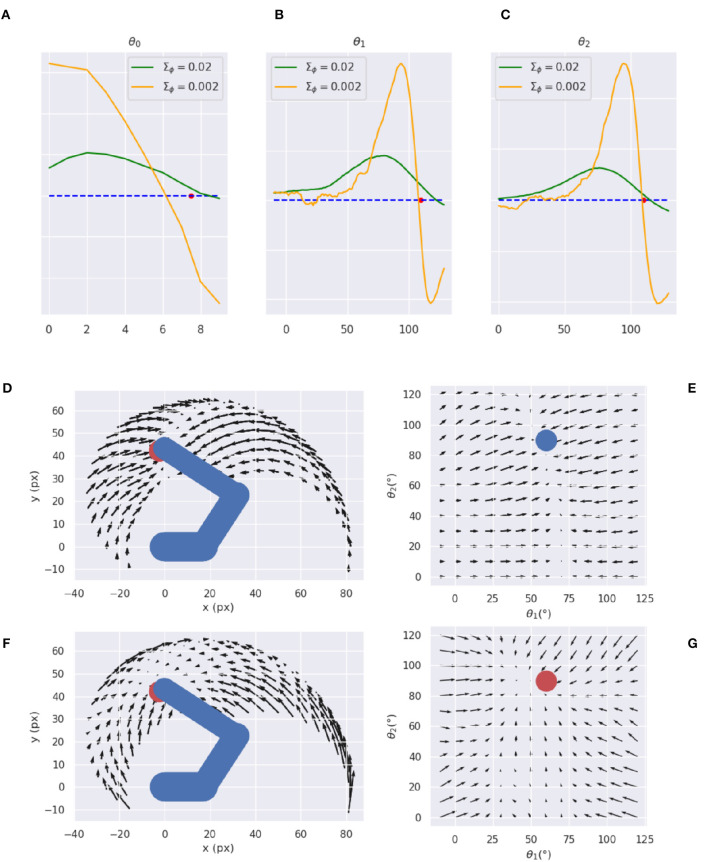
Visual gradient analysis. Marginal gradients for each joint, i.e., neck **(A)**, shoulder **(B)**, and elbow **(C)**, and for two values of the recognition density variance Σ_ϕ_ (green/orange line) computed by backpropagating the error between images with different arm configurations (abscissa: joint angle) and a reference image (whose angle is represented by the red dot on the abscissa). **(D–G)** Gradients for arm **(D, E)** and target **(F, G)** in both Cartesian **(D, F)** and joint **(E, G)** space.

Figures 14A–C reveals that a decoder with intermediate variance values (green line) causes smaller but smoother gradients, while a too-low variance (orange line) causes sharp peaks near the reference point and even incorrect gradient directions in some cases. Therefore, too low encoder variances seem to make the decoder prone to overfitting, while higher variance values help extract a smoother relationship between irregular multidimensional sensory domains and regular low-dimension causes. Figures 14D–G further illustrates the arm and target gradients relative to a sample reference posture and target location (the result is similar for other configurations) in both Cartesian and polar coordinates; the polar plot shows the two joints most relevant to the reaching action.

The plots reveal greater arm gradients (upper panels) in the vicinity of the target location; in that subspace, the decoder has less uncertainty about which direction to choose to minimize the error. Notably, the gradients tend to compose curved directions, a characteristic of biological motions. The polar plot provides critical insights into the causes of the circular pattern: the gradients are mostly parallel to the horizontal axis, which corresponds to a movement consisting essentially of pure shoulder rotation. Thus, they provide a strong driving force on the shoulder almost throughout the operational space, while the area in which the elbow is controlled is limited to the vicinity of the target location. These gradients result in a two-phase reaching of static targets in which the agent first rotates the shoulder— resulting in a horizontal positioning—and then starts to rotate the elbow as soon as the latter enters its attraction area. The same gradients can explain the linear motion pattern of an arm tracking dynamically moving targets when the arm is close to the target: in that case, all gradients provide motion force as explained above. On the other hand, the gradients of the target belief (bottom panels) behave somewhat differently: since this belief is unconstrained and can freely move in the environment, update directions more directly approach the target in all angular coordinates (see the polar plot to the right). Yet, linear belief updates in the polar space still translate to curve directions in the Cartesian space.

## 6. Discussion

We presented a normative computational theory based on Active Inference of how the neural circuitry in the PPC and DVS may support visually-guided actions in a dynamically changing environment. Our focus was on the computational basis of encoding dynamic action goals in the PPC through flexible motor intentions and its putative neural basis in the PPC. The theory is based on Predictive Coding (Doya, 2007; Hohwy, 2013), Active Inference (Friston, 2010), and evidence suggesting that the PPC performs visuomotor transformations (Cisek and Kalaska, 2010; Fattori et al., 2017; Galletti and Fattori, 2018) and encodes motor plans (Andersen, 1995; Snyder et al., 1997). Accordingly, the PPC is proposed to maintain dynamic expectations of both current and desired latent states over the environment and use them to generate proprioceptive predictions that ultimately generate movements through reflex arcs (Adams et al., 2013; Versteeg et al., 2021). In turn, the DVS encodes a generative model that translates latent state expectations into visual predictions. Discrepancies between sensory-level predictions and actual sensations produce prediction errors sent back through the cortical hierarchy to improve the internal representation. The theory unifies research on intention coding (Snyder et al., 1997) and current views that the PPC estimates the body and environmental states (Medendorp and Heed, 2019), providing specific computational hypotheses regarding the involvement in goal-directed behavior. It also extends some perception-bound Predictive Coding interpretations of the PPC dynamics (FitzGerald et al., 2015) and provides a more comprehensive account of movement planning (Erlhagen and Schöner, 2002), tightly integrated into the overall sensorimotor control process.

The core novelty with respect to state-of-the-art implementations in continuous time Active Inference is that we first considered an internal belief over not only bodily states but also every object in the scene, where the latter are encoded in the joint angles space as well, simulating a visuomotor reference frame that the PPC is supposed to encode. Then, we decomposed the belief dynamics into a set of independent intentions each depending on the current belief and predicting the next plausible state. Such formalization has several advantages. First, since attractors are dynamically generated at each time step, the agent can also follow moving targets and interact with a constantly changing environment, in contrast to static reaching tasks where a desired fixed state is specified in the belief dynamics (Baioumy et al., 2020). Second, expressing the target position in terms of a possible joint configuration—either imposed by higher levels for realizing specific affordances or freely inferred by the exteroceptive models—results in simple intentions, without the need to directly use sensory information or duplicating lower-level generative models, which leads to implausible scenarios (Lanillos et al., 2020; Sancaktar et al., 2020). It should be however noted that, although an intrinsic-only attractor is faster and more parsimonious, continuous activation of visual low-level attractors may provide more precise motor control. Indeed, it seems that motor areas are able, at a certain "neural energy" cost, to interact with and generate predictions in multiple sensory domains. The key difference is that in the former a single prediction—which is already biased toward a future state—is compared with the sensory input; in the latter, a prediction of the current state is compared with the desired exteroceptive goal, biasing the belief through the backpropagated gradient. Further studies are thus needed to implement low-level attractors in a biologically plausible way—e.g., intentions could generate through parallel pathways their own future sensory predictions that are compared with the observations in the usual way, with a particular intention that can be viewed as trying to continuously predict the current sensory input—and analyze the differences between the two modalities. Last, maintaining different belief components also allows easy encoding of previously memorized states which can be especially useful when implementing a sequence of actions, since only the intention precisions have to be adjusted. Indeed, it seems that the PPC explicitly encodes and maintains such goals during the whole unfolding of sequential actions (Baldauf et al., 2008). A specific goal is selected among other competitive intentions possibly under the control of the PFC and PMd (Stoianov et al., 2016) and fulfilled by setting it as a predominant belief trajectory as an attractor with a strong gain (see Equations 44, 26 and Figure 4). For example, in a typical reaching task, the goal of reaching a specific visual target corresponds to the future expectation that the agent's arm will be over that target; thus, if the agent maintains a belief over the latter, the corresponding intention links the expected belief over the future body posture with the inferred target, expressed in joint angles, encoding a specific interaction to realize.

We tested the computational feasibility of the theory on a delayed reaching task—a classical experiment in electrophysiology—in which a monkey is required to reach with its hand a visual-spatial target, starting the movement from an HB (Breveglieri et al., 2014). To do this, we simulated an agent consisting of a coarse 3-DoF limb model and noisy visual and proprioceptive sensors (Figure 3A). Simplified proprioceptive sensors provided a noisy reading of the state of the limb in joint angles, while visual input was provided by a fixed camera and consisted of an image of the target and limb. Predictive visual sensory processing simulating the DVS was implemented with a VAE trained to infer body state and target location, both in the joint angles domain (Figure 3C). The limbs were animated at the velocity level with motor control signals computed by the visually-guided Active Inference controller. The computational analysis showed, first and most importantly, that the controller could correctly infer the position of the visual targets (Figure 5, *t* = 70), use it to compute and set motor goals in terms of prior beliefs on the future body state through intention functions (Figure 5, *t* = 105), and perform adequate and smooth reach movements (Figure 5 *t* = 105–150), with and without visual feedback (Figure 6). The greater accuracy obtained with visual feedback parallels classical results in a similar classical behavioral comparison of reaching (Keele and Posner, 1968).

We then systematically investigated the effects of noise on various functional components (Figure 7), starting with the balance of the precision between proprioceptive and visual sensory models: a noiseless Active Inference agent (BL condition) resulted in the best performance, with a stable final approach and accuracy only limited by the quality of the visual target estimation. Among the noisy conditions, pure proprioceptive control resulted in the lowest performance, as expected. Motor control driven by both proprioceptive and visual feedback with balanced precision between the two domains resulted in improved reach accuracy and greatly improved arm belief stability (Figure 8). The effect on accuracy was mainly due to the inclusion of visual information in the inference process, but also to slower updates of the motor control signals due to decreased confidence about proprioceptive input. The increased stability of the arm belief did not improve movement stability as increasing confidence about visual input also increased the discrepancy between belief and action updates, the latter only relying on noisy proprioceptive observations. In fact, we showed that if we remove the plausibility constraint that motor control is driven only by proprioceptive predictions and thus let actions minimize prediction errors from all sensory domains, the reach performance greatly increases (Figure 9). Nonetheless, any combination of visual and proprioceptive feedback improved performance relative to a control driven by feedback from a single sensory domain. The instability due to the difference in update directions between belief and action could be balanced by other mechanisms that we have not considered here. For example, we assumed that the same pathway is used for both control and belief inference, but it seems that the motor cortex generates different predictions depending on the brain areas which it interacts with: purely proprioceptive predictions for motor control, whose prediction error is suppressed at the lowest level of the hierarchy, and rich somatosensory predictions for latent state inference, which integrates somatic sensations at different hierarchical levels (Adams et al., 2013). Intention precisions or attractor gains affected performance as well. First, they affected reach time: as expected, the greater the gain, the faster the movement. However, fast movements come at a cost: increased gains generally resulted in less precise movements and decreased stability during the final reach period. Finally, higher visual target precisions decreased perception time and improved perception accuracy but decreased perception stability.

We also investigated the effects of movement onset policies: response delay allows investigating perceptual and motor preparatory processes separately from the motor control and action execution. We found that delayed response decreased movement time with respect to a policy that requires an immediate response (Figure 10), which fits the behavioral pattern (Shenoy et al., 2013). Apparently, this is due to the need to estimate, in the latter condition, the target position “on the fly,” and constantly adapt the intention according to the updated target estimate. The advantage of allowing some preparatory time becomes clear in an anecdotal fly-catching task, which results in faster movement and increased chances of success. This comes with the critical contribution of PPC neurons that systematically modulate their activity during the preparatory period (Shenoy et al., 2013), which here provided specific predictions for the computations performed in the PPC. Notably, the immediate-response policy allowed the Active Inference controller to perform actions under dynamic environmental conditions, such as tracking moving objects. Free energy minimization resulted in rapid target detection also in this case, and maintained subthreshold perception error on moving targets (Figure 11) which allowed precise tracking after an initial reaching period.

Intention-driven Active Inference in continuous time largely compares to classical neural-level hypotheses of motor planning such as the Dynamic Field Theory (Erlhagen and Schöner, 2002), with the advantage of stepping on an established Predictive Coding framework, dynamic approximate probabilistic inference, and end-to-end sensorimotor control. The Dynamic Field Theory estimates the parameters of the desired movement—such as movement direction and target velocity—from sensory and task features encoding environmental descriptors, which closely compares to motor goal coding through flexible intentions in our model. The two theories have in common a dynamic activation of the internal representations in continuous time, governed by a dynamic system, but they differ in the nature of the signals and their coding. Movement descriptors in Dynamic Field Theory are represented by a dynamically activated multidimensional space, each encoded on a population of competing neurons, while Active Inference approximates movement properties with their central moments (belief) and dispersion (precisions). While population coding allows a complete description of a probabilistic distribution, it could be overshot when used to code single magnitudes (although it is essential when encoding discrete categories). Yet, such representation allows coding multiple competing targets on the same population of neurons, while in our scheme each target should be encoded by a dedicated unit. Notably, the brain encodes scalar variables using a variety of number coding schemes, including monotonic and distributed (Stoianov and Zorzi, 2012). The latter, known as a “mental number line” (Stoianov et al., 2008), could be an interesting hypothesis to explore also in the context of feature coding in continuous Active Inference. Currently, distributed coding is used only in discrete Active Inference and other probabilistic models to investigate computationally high-level cognitive functions such as planning, navigation, and control (Stoianov et al., 2016, 2022; Pezzulo et al., 2018). The two theories also differ in the nature of the input to their dynamic systems. In Active Inference, system input encodes generalized prediction errors, which are integrated into higher-level moments. Instead, input in Dynamic Field Theory directly encodes state values. Coding based on prediction errors has the advantage of minimizing the quantity of transmitted information—hence, energy. Finally, the theories also differ in scope: Active Inference provides a full account of the entire sensorimotor control process, while Dynamic Field Theory describes only movement planning.

### 6.1. Precision balance and conditions for disorders

**Figure 15:**
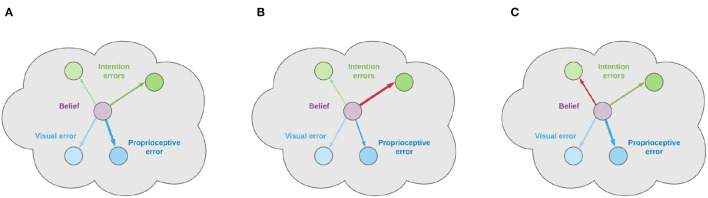
Normal and abnormal intention gains and precisions. Arrow widths represent strengths of prediction errors. In normal conditions, the sensory contribution to free energy minimization is bigger than that of the intentions and there is a clear unambiguous intention to fulfill **(A)**. An abnormally strong intention gain λ drives the belief away from the true joint states **(B)**. Too close precision levels γ also hinder implementing competing intentions **(C)**.

Based on our computational analysis, it becomes clear that some motor and behavioral disorders could be due to the lack of proper sensory and intention precisions (Adams et al., 2021). Here, we illustrate the normal condition and two types of potentially improper precision balance that could become a causal condition for neurological disorders. Figure 15A illustrates the condition for normal functioning, which is such that the contribution of a single intention to the belief update (which, as a reminder to the reader, is proportional to the gain of intentions λ) is sufficiently small with respect to the sensory contribution. In this case, during free energy minimization, the system dynamics smoothly moves the belief toward the strongest goal, along with precise tracking of the true latent state and sensory signal of the limbs, allowing thus to compute correct motor control errors and perform smooth action execution. A critical abnormal condition arises when the intention gain λ is too strong, as illustrated in Figure 15B. In this case, the belief moves too rapidly toward the goal without being able to match the proprioceptive observations, which results in computing incorrect motor control signals. Another abnormal condition is caused by too close precisions ***γ***_*k*_ of competitive intentions, which is likely to result in opposing belief updates and thus prevent the fulfillment of any of the competing goals (as in Figure 15C). This situation might manifest in terms of motor onset failure or oscillatory behavior.

### 6.2. Neural-level predictions

One peculiarity of Active Inference based theories of motor control is that proprioceptive predictions are sent through efferents down to the spinal cord and that specific muscle control signals are computed at that level by reflex arcs, so that action attempts to suppress proprioceptive prediction errors (Adams et al., 2013). This prediction critically differs from competing modern theories such as the Optimal Control (Todorov and Jordan, 2002), according to which the efferents convey muscle control signals computed at the cortical level. A general aspect of Active Inference regards the dynamic inferential process, which predicts with increasing precision the internal representation of the sensorium—including estimation of targets and body posture—starting from noisy priors that gradually converge to ideal states. This kind of precision trend should be observed in an experiment with multiple repetitions of the same action and target, with variability of cell activity encoding the target and body that gradually decreases in time within trials. While this prediction is generally shared with Predictive Coding based theories, classical stimulus-response theories would predict invariant variability of cell activity across time. Another general aspect regards coding of prediction errors. In fact, body-environment transitions involving a change of states and tasks result in transient bursts of activity in error-conveying cells until the error is minimized. Prediction errors conveying upstream information are supposed to be encoded by pyramidal cortical cells in superficial layers while downstream predictions are encoded by deep pyramidal neurons (Parr et al., 2022).

In light of the considerations so far, we predict several different types of correlates that should be found in the PPC related to coding environment, task, and bodily states. The former two include correlates of potential spatial targets and selected motor goals, which indeed have been consistently found in the PPC (Andersen, 1995; Snyder et al., 1997; Filippini et al., 2018). The latter includes correlates of intention-biased bodily state estimates, which thus are not precise representations of the true states. To this concern, a key expected neural correlate of the proposed mechanism in the PPC includes signals encoding intention prediction errors between the current belief and future states corresponding to targets to interact with, both encoded in a visuomotor reference frame. To investigate this, one can manipulate high-level priors, e.g., by inducing an abrupt change of the intention, which should then be observed as a fast decaying change of the corresponding prediction error. A related hypothesis is that in tasks comprising several targets—like the classical monkey experiment analyzed here—each goal generates its own intention and prediction error. In normal conditions only one intention is selected at a time, and this behavior should be observed in the relative dynamics between all the intention prediction errors encoded simultaneously. Finally, the use of generalized beliefs in Active Inference predicts that the PPC encodes not only static states but also a detailed estimate of body dynamics, up to a few temporal orders. Indeed, a body of literature report motion-sensitive, or Vision-for-Action activity in the DVS and PPC (Galletti and Fattori, 2018). The validation of all these correlates will be the subject of further studies with real monkey experiments similar to the one described in Figure 3B.

### 6.3. Limitations and future directions

Our focus here was on intention coding in the PPC, which directly deals with motor plans and motor control. Further elaborations will extend the theory with higher-level aspects of cognitive control, including intention structuring (dealt by PM), phasing (SMA) (Gallego et al., 2022), planning, and goal selection (HC, PFC) (Stoianov et al., 2016, 2018; Pezzulo et al., 2019). Motor control operated here in an inner belief space belonging to the joint angles domain, which is generally suboptimal in the external Cartesian space. Although a kinematic transformation was implicitly performed by the VAE, we assumed that neural activity in the PPC encodes generalized beliefs over targets and body only in a motor-related domain; however, neural data suggest that neurons in the motor cortex encode motor trajectories also in extrinsic coordinates (Cohen and Andersen, 2002; Adams et al., 2013), and a more realistic model should include representations encoding states in both intrinsic and extrinsic reference frames. A functional correlate of the motor cortex should represent future states—which were defined here implicitly in the intention prediction errors and dynamics functions—and transform desired trajectories from Cartesian coordinates to proprioceptive predictions in the intrinsic state-space. This transformation is different from Optimal Control planning since the optimization of a classical inverse model reduces to a more manageable inference problem.

Since our focus was on the theoretical introduction of intentionality in Active Inference, every analysis was only partially characterized by a simple reaching task. However, fundamental properties of the physical model, including geometry, mass, and friction, strongly influence the resulting motion dynamics—hence the entire inferential process. This implementation does not adopt other important neural and biomechanical specificities such as signal delay and joint friction (Wolpert and Flanagan, 2016), and just partially covers the three main domains of sensorimotor learning through a predictive forward control; for example, it does not fully include reactive, stimuli-driven control such as obstacle avoidance, although we showed that it can successfully perform static and dynamic tasks. However, it could be easily extended to accommodate additional sensory modalities—e.g., tactile sensations—with rich generative models such as the VAE implemented here. Further planned computational analyses will use a richer belief space, a more realistic physical arm model, and additional actuators, and expand the complexity of the intention functions to investigate the capacity of the theory to explain in-depth neural levels, cognitive, and kinematic phenomena related to motor learning, motion perception, motor planning, and so on. Planned future studies with a more articulated agent will also challenge the theory at the behavioral and neural level against other empirical findings regarding movement preparation and motor control, in either delayed or direct response settings. For example, we will test the model for stimulus-stimulus congruency and stimulus-response compatibility effects (Kornblum et al., 1990). As for the former, it is intuitive that a greater sensory dimension overlap predicts faster target-belief convergence—thus faster intention setting. Less intuitive is that stimulus-response compatibility effects should emerge due to differences in the dynamic transition from one belief state to another in the proprioceptive domain. For example, a belief over the effector state should change more, requiring more time to converge when reaching a contralateral position than an ipsilateral one.

Although we considered an Active Inference model with just a single layer of intentions, the structure represented in Figure 2 could be scaled hierarchically and intermediate goals could be considered between high-level intentions and low-level sensory generative models, e.g., by combining discrete and continuous Active Inference for planning and movement execution (Friston et al., 2017a,b; Parr et al., 2020; Sajid et al., 2021). According to the free energy principle, the agent will then choose goals and subgoals and rely on specific sensory modalities such that free energy is minimized at every hierarchical level based on prediction errors coming from the level below. This formalization will provide an explicit basis for motor planning, including tasks like object manipulation. Indeed, although the current implementation performs well on spatial tasks like reaching in a dynamically changing environment, it cannot implement composite goals which the brain needs to handle. On the other hand, an agent that can encode higher-level goals in a discrete domain and infer policies based on the *expected free energy* will be able to dynamically modify its behavior and react to environmental changes. An extended implementation of this kind—showing the interplay between discrete goals and continuous intentions—will be the subject of future work.

## Data availability statement

Publicly available datasets were analyzed in this study. This data can be found at: https://github.com/priorelli/PACE.

## Author contributions

MP developed the computational method, wrote the code, run the simulations, analyzed the results, and wrote the draft. IS developed theoretical and methodological ideas and wrote the draft. All authors contributed to the article and approved the submitted version.

## Footnotes

^1^Python code provided in https://github.com/priorelli/PACE.
